# CCL5^hi^ Macrophages Interact with CD8^+^ T Cells and Potentiate Responsiveness to PD‐1 Blockade Plus Chemotherapy in Esophageal Squamous Cell Carcinoma

**DOI:** 10.1002/advs.202409187

**Published:** 2025-09-25

**Authors:** Yuanzhen Ma, Xiaodong Su, Tingting Zeng, Chunlian Zhou, Guozhen Yang, Zengfei Xia, Chufeng Zeng, Jiarong Zhan, Xin‐Yuan Guan, Xu Zhang, Yan Li

**Affiliations:** ^1^ State Key Laboratory of Oncology in South China Guangdong Provincial Clinical Research Center for Cancer Sun Yat‐sen University Cancer Center Guangzhou 510060 P. R. China; ^2^ Guangdong Esophageal Cancer Institute, Department of Thoracic Oncology Sun Yat‐sen University Cancer Center Guangzhou 510060 China; ^3^ Department of Clinical Oncology The University of Hong kong Hong Kong China

**Keywords:** CCL5, CD8^+^ T cells, esophageal squamous cell carcinoma, immunochemotherapy, macrophages, PD‐1

## Abstract

Immunotherapy has significantly transformed cancer therapy and reinvigorated research in cancer immunology. Several clinical trials have employed neoadjuvant immunochemotherapy (NIC) for advanced esophageal squamous cell carcinoma (ESCC), resulting in significant benefits for many patients. However, response rates remain limited, underscoring the need for more effective screening methods and novel therapeutic strategies. Tumor‐associated macrophages (TAMs) have been shown to play a crucial role in tumor progression and impact the clinical outcomes of cancer patients. Nonetheless, it is worth noting that TAMs exhibit phenotypic and functional heterogeneity. In this study, a distinct population of CCL5^hi^ macrophages is identified that plays a critical role in ESCC when treated with immunochemotherapy. Furthermore, insights are gained into the underlying mechanisms, revealing the existence of a feedback loop involving IFN‐γ, CCL5^hi^ macrophages, and CD8^+^ T cells within the tumor microenvironment of ESCC undergoing immunochemotherapy. Specific signatures associated with CCL5^hi^ macrophages are correlated with a better response to immunochemotherapy in patients with ESCC. Moreover, the in vivo experiments demonstrate that CCL5 supplementation enhances the effectiveness of immunochemotherapy. This research contributes to the optimization of patient stratification and provides a rationale for novel combinatorial approaches involving immunotherapy in the treatment of ESCC.

## Graphical abstract

This study has characterized a functionally distinct macrophage subset, termed CCL5hi macrophages, and elucidated the underlying mechanisms of a regulatory feedback loop that interconnects IFN‐γ, CCL5hi macrophages, and CD8+ T cells within the TME of ESCC undergoing immunochemotherapy. Notably, exogenous administration of CCL5 significantly enhances the in vivo therapeutic efficacy of immunochemotherapy.

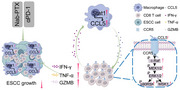

## 1 Introduction

Esophageal cancer is a significant global health issue, with more than 500,000 newly diagnosed cases annually, accounting for over 2.5% of all cancer cases and ranking seventh in terms of cancer‐related mortality rates.^[^ ^1^ ^]^ Esophageal squamous cell carcinoma (ESCC) is the most prevalent subtype worldwide, particularly in high‐risk areas for esophageal cancer.^[^ ^2^ ^]^ Because most ESCC patients are diagnosed with locally advanced tumors with lymph node metastasis, poor prognosis remains the major challenge for ESCC.^[^ ^3^ ^]^

Neoadjuvant chemotherapy^[^ ^4^ ^]^ or neoadjuvant chemoradiotherapy^[^ ^5^, ^6^ ^]^ are common strategies that have been shown to improve the prognosis of patients with advanced ESCC. Recently, the advances of immunotherapy have revolutionized cancer therapy and reinvigorated research in cancer immunology.^[^ ^7^ ^]^ Immune checkpoint inhibitors (ICIs), such as anti‐PD‐1 (anti‐Programmed cell death 1),^[^ ^8^, ^9^ ^]^ anti‐PD‐L1 (anti‐programmed death ligand 1)^[^ ^10^ ^]^ and anti‐CTLA‐4 (anti‐cytotoxic T lymphocyte‐associated protein 4),^[^ ^8^, ^10^ ^]^ have demonstrated durable clinical responses across multiple types of cancer. PD‐1 antibody plus chemotherapy has been shown to significantly improve overall survival and progression‐free survival in patients with advanced or metastatic ESCC when compared to placebo plus chemotherapy.^[^ ^11^, ^12^, ^13^ ^]^ Moreover, many clinical trials of neoadjuvant immunochemotherapy (NIC) (neoadjuvant immune checkpoint blockade combined with chemotherapy) have been applied to advanced ESCC, with many patients benefiting from the regimens.^[^ ^14^, ^15^, ^16^ ^]^ However, the remarkable responses to immunotherapies are currently limited to a minority of patients, highlighting the need for more effective approaches to screen patients and develop novel therapeutic strategies.

The tumor‐immune ecosystem is highly complex and consists of a heterogeneous collection of cells. Immune infiltrates in the tumor microenvironment (TME) have been shown to play a crucial role in tumor progression and impact the clinical outcomes of cancer patients.^[^ ^7^ ^]^ Although significant efforts have been dedicated to investigating T cells, other immune cells, including macrophages, dendritic cells, NK cells, and B cells, have also been demonstrated to contribute to tumor progression and responses to immunotherapy.^[^ ^7^ ^]^ Macrophages, as phagocytic cells, represent a heterogeneous population with complex phenotypic and functional properties within the TME.^[^ ^17^ ^]^ Specific subsets of tumor‐associated macrophages may have distinct roles in tumor progression and antitumor immunity. For example, the density of FOLR2^+^ macrophages in breast cancer positively correlates with improved patient survival.^[^ ^18^ ^]^ Furthermore, targeted therapeutic interventions focused on macrophage subsets have been explored. CD40‐mediated activation of macrophages has been shown to enhance the response to anti‐PD‐1 therapy in intrahepatic cholangiocarcinomas.^[^ ^19^ ^]^ Additionally, reprogramming macrophages from M2‐like immunosuppressive macrophages to M1‐like immunostimulatory macrophages using a BCL‐2 inhibitor has been shown to restore T cell function and promote a favorable response to immunotherapy.^[^ ^20^ ^]^

Here, we have identified a functionally distinct macrophage subset, termed CCL5^hi^ macrophages, that plays a critical role in ESCC treated with immune checkpoint blockade (ICB) plus chemotherapy. Furthermore, we have uncovered the underlying mechanisms of a feedback loop involving IFN‐γ, CCL5^hi^ macrophages, and CD8^+^ T cells within the TME of ESCC treated with immunochemotherapy. Importantly, specific signatures associated with CCL5^hi^ macrophages are correlated with a favorable response to immunochemotherapy in patients with ESCC. Additionally, supplementation of CCL5 enhances the therapeutic efficacy of ICB plus chemotherapy in in vivo experiments. Our findings have significant implications for optimizing patient stratification and provide a rational for novel combinatorial approaches with immunotherapy in the treatment of ESCC.

## 2 Results

### 2.1 CD8^+^ T Cells and Macrophages Play Critical Roles in ESCC Patients Receiving NICB Therapy

**Figure 1:**
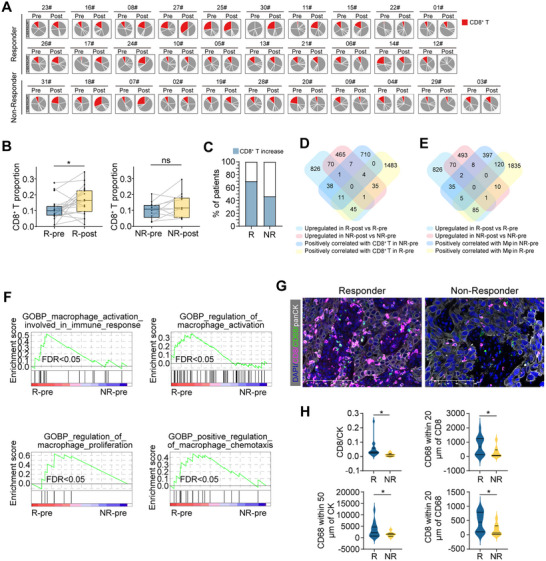
CD8^+^ T cells and macrophages in ESCC receiving NICB therapy. A) Pie chart showing the proportion of CD8^+^ T cells in immune cells in ESCC tissues (pre‐therapy and post‐treatment) receiving NICB therapy (n = 31; SYSUCC cohort). B) CD8^+^ T cells proportion in immune cells in paired pre‐therapy and post‐treatment tissues of responders (n = 20) or non‐responders (n = 11) (paired *t*‐test). C) The percentage of patients with CD8^+^ T cells increases after NICB therapy in responder and non‐responder groups. D,E) Venn diagram of the number of genes based on differential gene expression analyses or correlation analyses with CD8^+^ T cells (D) and macrophages (E). F) Gene set enrichment analysis plots showing the signaling pathways in a ranked list of genes (SYSUCC cohort). G) Representative multiplex images of CD8^+^ T cells (CD8^+^), macrophages (CD68^+^), and tumor cells (panCK^+^) in a responder patient or non‐responder patient before NICB therapy (SYSUCC cohort). H) Density or spatial distribution of CD8^+^ T cells, macrophages, or tumor cells in responder and non‐responder groups (SYSUCC cohort). (pre, pre‐therapy; post, post‐treatment; R, responders (n = 17); NR, non‐responders (n = 9); Student's *t*‐test; ^*^ *p* <0.05).

Tumor tissues were collected from 31 patients with ESCC receiving neoadjuvant PD‐1 blockade (camrelizumab) combined with chemotherapy (nab‐paclitaxel and capecitabine).^[^ ^15^ ^]^ Patients were divided into responder and non‐responder groups according to their response to the combined therapy: patients with tumor regression≥90% were defined as responders (R) and tumor regression<90% as non‐responders (NR).^[^ ^15^ ^]^ Tissues were collected before and after the neoadjuvant therapy separately, and bulk RNA sequencing was performed on these samples. The immune cell infiltration was analyzed by online software (TIMER 2.0) (http://timer.cistrome.org/). The results showed that the proportion of CD8^+^ T cell infiltration was increased in post‐treatment tissues compared with pre‐therapy tissues in the responder group (R) (*P*<0.05), whereas no significant difference was observed in the non‐responder group (NR) (**Figure** **1**A,B). Additionally, no significant difference in the proportion of CD8^+^ T cells was observed in pre‐therapy tissues between the R group and the NR group (Figure S1, Supporting Information). The increase in CD8^+^ T cells proportion was observed in 14 ESCC patients in the responder group (20 cases, 70%) after neoadjuvant therapy, and only 5 in 11 cases (45%) in the non‐responder group (Figure 1C).

Next, the RNA‐seq data were screened, and genes upregulated after treatment in the responder group were intersected with genes positively correlated with CD8^+^ T cell or macrophage infiltration. This generated 45 genes related to CD8^+^ T cell infiltration and 85 genes related to macrophage infiltration (Figure 1D,E). These results indicate that macrophages might also play important roles in patients’ responses to the combined treatment. In addition, gene set enrichment analysis (GSEA) results displayed that pathways related to macrophage activation, proliferation or chemotaxis were significantly enriched in the responder group (Figure 1F).

To further investigate the spatial correlation between CD8^+^ T cells and macrophages, multiplex fluorescent immunohistochemistry (mfIHC) was carried out on 26 pre‐therapy tumor tissues, including 17 cases in the responder group and 9 cases in the non‐responder group. The ratio of CD8^+^/panCK^+^ cells was found to be significantly increased in the responder group compared to the non‐responder group (*P*<0.05, Figure 1G,H). Additionally, when calculating the intercellular distance, CD68^+^ macrophages were significantly enriched within 50 µM of panCK^+^ tumor cells in the responder group compared to the non‐responder group. Furthermore, CD68^+^ macrophages were significantly enriched within 20 µM of CD8^+^ T cells, and vice versa (*P*<0.05, Figure 1G,H). These findings suggest a spatial correlation between CD8^+^ T cells and macrophages within the tumor microenvironment and highlight their potential interaction in response to the combined treatment.

**Figure 2:**
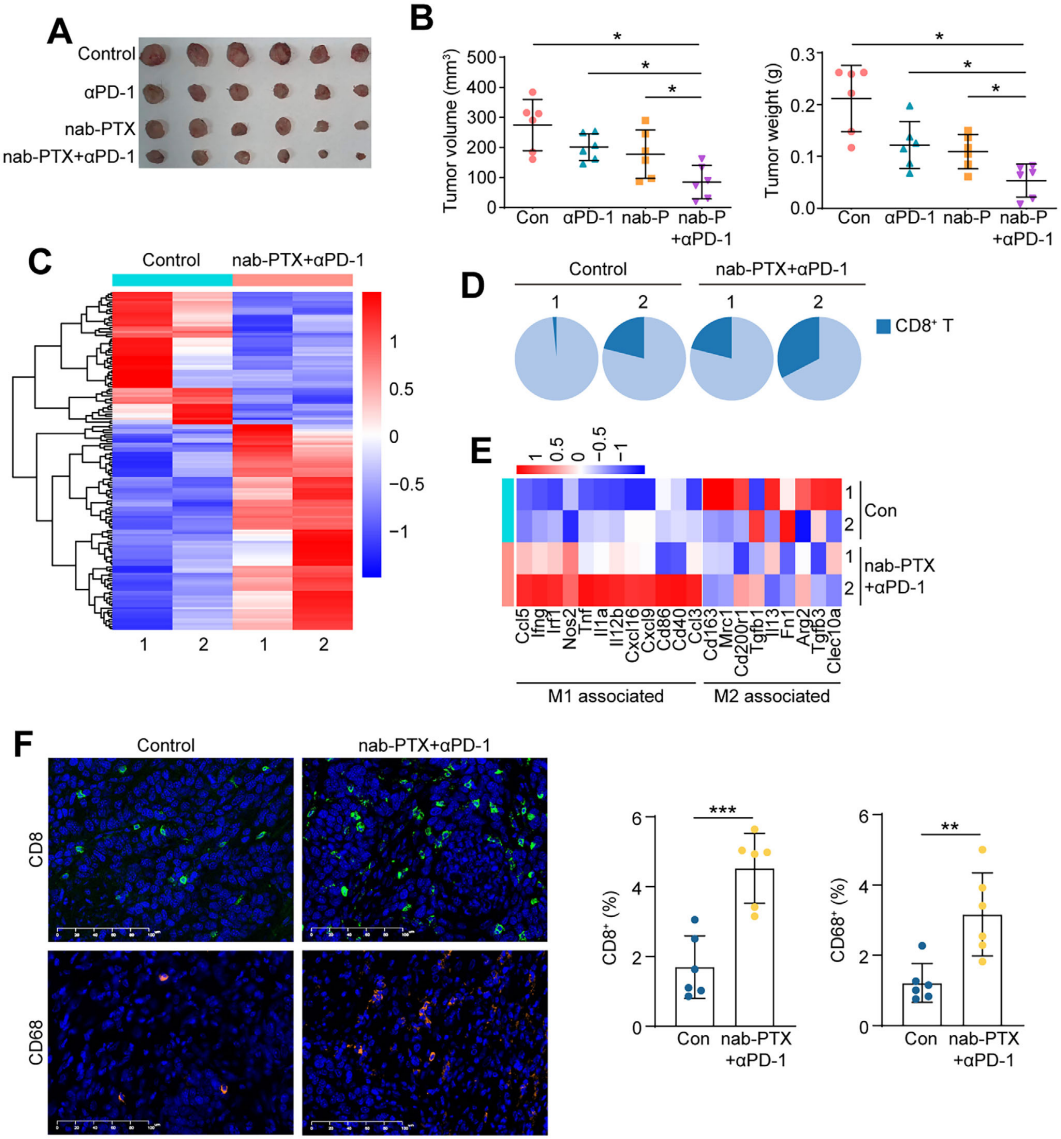
Immunochemotherapy (nab‐PTX+anti‐PD‐1) suppresses tumor growth in vivo. A) Images of syngeneic tumors formed on C57BL/6J mice that were treated with anti‐PD‐1 (αPD‐1), nab‐PTX, nab‐PTX+anti‐PD‐1, and vehicle control (n = 6 per group). B) Tumor volume (*left*) and tumor weight (*right*) of (A) were summarized (n = 6 per group; Student's *t*‐test). C) Heatmap of top 150 DEGs between nab‐PTX+anti‐PD‐1 samples and control samples (n = 2 per group). The significance was analyzed by DESeq2. D) Pie chart showing the proportion of CD8^+^ T cells in immune cells based on RNA‐seq data of nab‐PTX+anti‐PD‐1 and control samples (n = 2 per group). E) Heatmap showing the expression of M1‐type associated genes or M2‐type associated genes in nab‐PTX+anti‐PD‐1 and control samples. F) Representative images and summary of IF staining of CD8 and CD68 in tumors of nab‐PTX+anti‐PD‐1 and control groups (n = 6 per group; Student's *t*‐test). (^*^ *p* < 0.05; ^**^ *p* <0.01; ^***^ *p* <0.001).

To assess the therapeutic efficacy of the combined therapy in vivo, a mouse model was established by inoculating mEC2, a mouse esophageal squamous cell carcinoma cell line, subcutaneously into immune competent C57BL/6J mice. Tumors were then sliced and implanted into additional C57BL/6J mice. The animals were divided into different treatment groups, including mPD‐1 antibody, nab‐PTX, mPD‐1+nab‐PTX (combo), and a blank control group. The results showed that the tumor growth was significantly inhibited by the combined therapy (*P*<0.05; **Figure** **2**A,B; Figure S2A, Supporting Information). Tumors were isolated from the combo group and the control group (n = 2 per group), and bulk RNA‐seq was performed. Analysis of the top 150 differentially expressed genes (DEGs) using GO (gene ontology) and KEGG (kyoto encyclopedia of genes and genomes) analyses revealed significant enrichment of gene signatures related to cellular response to interferon‐gamma (IFN‐γ), positive regulation of T cell proliferation, and PD‐L1 expression and PD‐1 checkpoint pathways in the combo group compared to the control group (Figure 2C; Figure S2B,C, Supporting Information). The proportion of CD8^+^ T cell infiltration was increased in the combo group (Figure 2D). The expression of select markers associated with M1 and M2 macrophages was also compared, and it was found that M1‐associated molecules, such as IFN‐γ, were increased in the combo group, while M2‐associated molecules, such as CD163, were decreased (Figure 2E). Immunostaining for CD8 and CD68 confirmed that infiltration of CD8^+^ T cells and macrophages was increased in the tumor tissues of the combo group (Figure 2F; Figure S3, Supporting Information). These findings provide further evidence that CD8^+^ T cells and macrophages play important roles in the response to the combined immunochemotherapy.

### 2.2 Macrophage Depletion Reduces the Therapeutic Effectiveness of Immunochemotherapy

**Figure 3:**
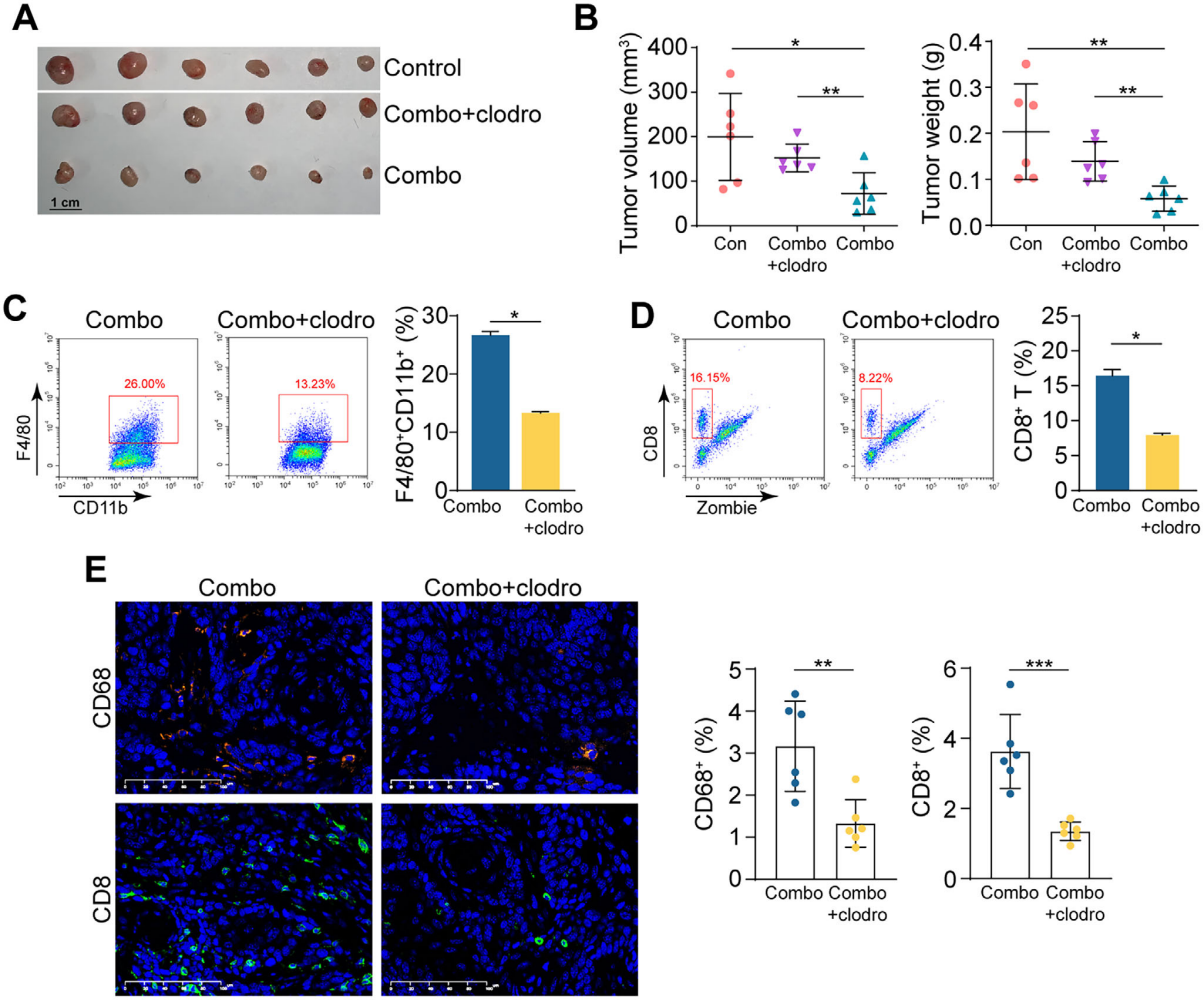
Depletion of macrophages attenuates the therapy effect of immunochemotherapy. A) Image of syngeneic tumors formed on C57BL/6J mice that were treated with nab‐PTX+anti‐PD‐1 (combo), nab‐PTX+anti‐PD‐1+clodronate (combo+clodro), and vehicle control (n = 6 per group). B) Tumor volume (*left*) and tumor weight (*right*) of (A) were summarized (n = 6 per group; Student's *t*‐test). C,D) Representative plots and quantification of F4/80^+^CD11b^+^ macrophages (C) and CD8^+^ T cells (D) in tumors of combo and combo+clodro groups using flow cytometry (n = 3 per group; Student's *t*‐test). E) Representative images and summary of IF staining of CD68 and CD8 in tumors of combo and combo+clodro groups. (n = 6 per group; Student's *t*‐test) (^*^ *p* < 0.05; ^**^ *p* < 0.01; ^***^ *p* < 0.001).

To further investigate the role of macrophages in the response of ESCC patients to immunochemotherapy, we depleted macrophages in mice using clodronate liposomes. The depletion was carried out either before or one week after implanting tumors. Following macrophage depletion, the animals were treated with PD‐1 blockade and chemotherapy. Similar to previous results, macrophage depletion resulted in a significant inhibition of therapeutic effectiveness in the clodronate‐treated groups (**Figure** **3**A,B; Figure S4A–D, Supporting Information). The tumors were isolated, digested, and stained, flow cytometry analysis demonstrated a significant decrease in F4/80^+^CD11b^+^ cells in the clodronate‐treated group (Figure 3C). This was further confirmed by immunofluorescence (IF) staining, which showed a reduction in CD68^+^ cells in the clodronate‐treated group (Figure 3E). Notably, CD8^+^ T cells were also reduced in the clodronate‐treated group (Figure 3D,E). To rule out the possibility of non‐specific effects of clodronate,^[^ ^21^ ^]^ CSF1R (colony‐stimulating factor 1 receptor) antibody was used to deplete macrophages in the in vivo assay. Similar results were observed, and the therapeutic effect was diminished by the use of anti‐CSF1R (Figure S4E–H, Supporting Information). Taken together, these results validate the importance of macrophages in the response to immunochemotherapy and highlight their interaction with CD8^+^ T cells in the tumor microenvironment.

### 2.3 Single‐Cell RNA Sequencing Reveals CCL5^hi^ Macrophage Subset

To elucidate the complexity of cellular compositions within the tumor microenvironment of ESCC receiving immunochemotherapy, we employed single‐cell RNA‐seq (scRNA‐seq) on unsorted cells derived from CD45‐positive cells in tumors of C57BL/6J mice treated with immunochemotherapy (combo) or vehicle (control) (Figure 2A). The mean number of reads per cell was 41,327 for the control sample and 44,204 for the combo sample. After filtering out low‐quality cells, a total of 7,763 cells from the control sample and 8,068 cells from the combo sample were retained for further analysis, which detected a median of 1,327 genes per cell in the control group and 1,571 genes per cell in the combo group.

**Figure 4:**
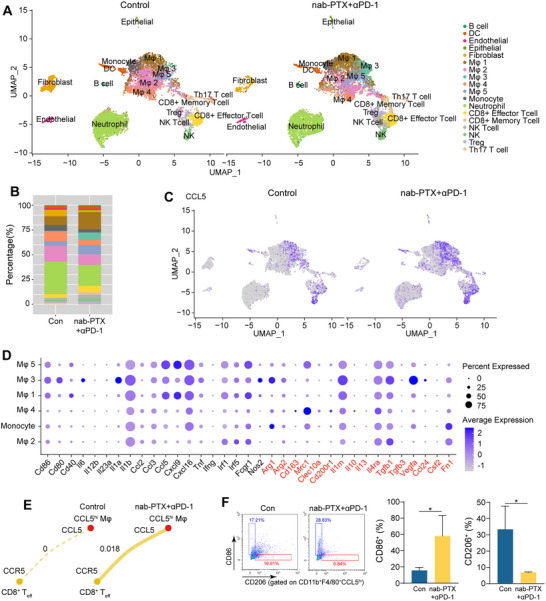
Single‐cell RNA sequencing reveals CCL5^hi^ macrophage subsets. A) UMAP plot visualization showing all CD45^+^ cell clusters from tumors of nab‐PTX+anti‐PD‐1 and control groups. Cells are colored by their cellular identity. B) Percentages of different cell clusters in tumors of nab‐PTX+anti‐PD‐1 and control groups. C) UMAP plot visualization of CCL5 expression in cell clusters from tumors of nab‐PTX+anti‐PD‐1 and control groups. D) Bubble plot showing the expression of M1‐ or M2‐associated molecules in different macrophage subsets and monocytes. E) Cell interaction strength of ligand‐receptor pair from CCL5 (CCL5^hi^ macrophages) to CCR5 (CD8^+^ T_eff_ cells) in tumors of nab‐PTX+anti‐PD‐1 and control groups. F) Representative plots and quantification of CD86 or CD206 in CCL5^hi^F4/80^+^CD11b^+^ cells from tumors of nab‐PTX+anti‐PD‐1 and control groups (n = 3 per group; Student's *t*‐test). (^*^ *p* < 0.05).

The UMAP (uniform manifold approximation and projection) analysis partitioned the cells into 18 clusters in both the control and combo samples based on marker genes (**Figure** **4**A; Table S1, Supporting Information). Macrophages were assigned to 5 cell clusters (Figure 4A,B), with an increase in the proportions of Macrophages 1, 3 and 5 in the combo sample compared with the control: Macrophage 1 (17.38% in combo vs. 8.82% in control); Macrophage 3 (7.66% in combo vs. 0.73% in control), and Macrophage 5 (9.02% in combo vs. 4.50% in control) (Figure 4B). Conversely, the proportions of Macrophage 2 and 4 were decreased in the combo compared with the control: Macrophage 2 (10.60% in combo vs. 16.14% in control) and Macrophage 4 (5.35% in combo vs. 9.71% in control). The differential expression analysis of macrophage clusters 1, 3, and 5 from the combo and control groups revealed that *CCL5* was the most upregulated gene in Macrophage clusters 1, 3, and 5 in the combo compared with the control (Figure 4C). Analysis of the expression of M1‐ and M2‐associated molecules indicated that macrophage clusters 1, 3, and 5 were more likely M1‐type macrophages (Figure 4D). The three macrophage clusters 1, 3, and 5 were integrated and named “CCL5^hi^ macrophages”. Cell communication patterns were identified using CellChat analysis, which detected six significant ligand‐receptor pairs between CCL5^hi^ macrophages and CD8^+^ effector T cells in the combo group (Table S2, Supporting Information). Notably, CCL5‐CCR5 was the most important interaction between CCL5^hi^ macrophages and CD8^+^ T_eff_ cells in the combo group, while no interaction was observed in the control group (Figure 4E). To further validate our findings, we isolated CCL5^hi^CD11b^+^F4/80^+^ cells from tumors of animals treated with immunochemotherapy or vehicle. Flow cytometry analysis revealed an increase in CCL5^hi^ macrophages (CCL5^hi^CD11b^+^F4/80^+^) among viable cells isolated from tumors of the immunochemotherapy group relative to the control group (Figure S5, Supporting Information). Additionally, an increase in CD86 (M1‐related marker) expression and a decrease in CD206 (M2‐related marker) expression were observed in the CCL5^hi^ macrophages of the immunochemotherapy group compared to the control group (Figure 4F). These findings further support the role of CCL5^hi^ macrophages in response to the immunochemotherapy.

### 2.4 CCL5^hi^ Macrophages Interact with CD8^+^ T Cells

**Figure 5:**
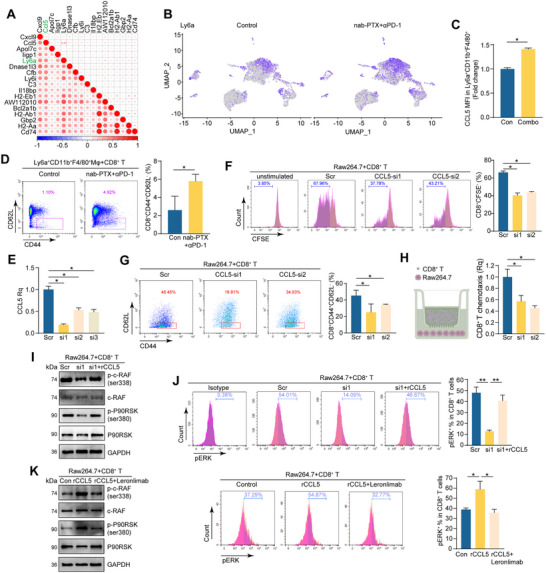
CCL5^hi^ macrophages activate CD8^+^ T cells. A) Correlation map analyzing the association of CCL5 to genes of surface proteins in the scRNA‐seq data. B) UMAP plot visualization of Ly6a expression in cell clusters from tumors of nab‐PTX+anti‐PD‐1 and control groups. C) The relative quantification of MFI of CCL5 in Ly6a^+^CD11b^+^F4/80^+^ cells from tumors of nab‐PTX+anti‐PD‐1 and control groups (n = 3 per group; Student's *t*‐test). D) Representative flow cytometry plots and summary of CD8^+^CD44^+^CD62L^−^ ratio in CD8^+^ cells co‐cultured with Ly6a^+^CD11b^+^F4/80^+^ macrophages from tumors of nab‐PTX+anti‐PD‐1 and control groups (n = 3 per group; Student's *t*‐test). E) RNA levels of CCL5 in RAW264.7 cells transfected with siRNAs targeting CCL5 (n = 3 per group; Student's *t*‐test). F) CD8^+^ T cells were co‐cultured with RAW264.7‐CCL5‐siRNAs or control cells in the presence of IL‐2. Representative flow cytometry plots and CD8^+^CFSE^−^/CD8^+^ ratio were summarized (n = 3 per group; Student's *t*‐test). G,H) CD8^+^ T cells were co‐cultured with RAW264.7‐CCL5‐siRNAs or control cells. Representative flow cytometry plots and summary of CD8^+^CD44^+^CD62L^−^/CD8^+^ ratio (G) (n = 3 per group; Student's *t*‐test), experimental design of chemotaxis, and relative quantification of chemotaxis of CD8^+^ T cells (H) (n = 3 per group; Student's *t*‐test). I) CD8^+^ T cells were co‐cultured with RAW264.7‐CCL5‐siRNA or control cells. Then, cells were supplemented with rCCL5 (recombinant CCL5 protein). The protein levels of CD8^+^ T cells were measured. GAPDH was a loading control. J) Representative flow cytometry plots and summary of pERK^+^CD8^+^ / CD8^+^ cells ratio (n = 3 per group; Student's *t*‐test). K) CD8^+^ T cells were co‐cultured with RAW264.7 cells in the presence of recombinant CCL5 protein, with or without the addition of leronlimab. The protein levels of CD8^+^ T cells were measured. (^*^ *p* < 0.05; ^**^ *p* < 0.01).

Because CCL5 is not a membrane protein expressed on cells, we further explored the scRNA‐seq data to look for surface proteins that correlate positively with CCL5 in macrophages/monocytes. Among the surface proteins associated with CCL5, Ly6a exhibits one of the highest correlation coefficients (**Figure** **5**A,B). To validate this finding, Ly6a^+^CD11b^+^F4/80^+^ cells were isolated from tumors of the combo and control groups using fluorescence‐activated cell sorting (FACS), and CCL5 expression was measured. Flow cytometry analysis revealed that CCL5 was increased in Ly6a^+^CD11b^+^F4/80^+^ macrophages in the combo group compared to the control group (Figure 5C; Figure S6, Supporting Information).

To investigate the functional significance of CCL5^hi^ macrophages in promoting CD8^+^ T cell activity, CD8^+^ T cells from healthy C57BL/6J mice were co‐cultured with Ly6a^+^CD11b^+^F4/80^+^ macrophages. The proportion of CD8^+^ T effector cells (CD8^+^CD44^+^CD62L^−^) was significantly increased in CD8^+^ T cells co‐cultured with macrophages from the combo group compared to those co‐cultured with macrophages from the control group (n = 3) (Figure 5D). To further investigate the impact of CCL5 expression in macrophages on CD8^+^ T cells, we transfected RAW264.7 (a mouse monocytic cell line) with siRNAs targeting CCL5 (Figure 5E) and co‐cultured these cells with CD8^+^ T cells. CFSE staining demonstrated that the proliferation of CD8^+^ T cells was reduced when co‐cultured with macrophages transfected with CCL5 siRNAs compared to those co‐cultured with scramble control macrophages (Figure 5F). Furthermore, CD8^+^ T cells derived from OT1 mice were co‐cultured with Raw264.7‐CCL5‐KD cells in the presence of the SIINFEKL OVA peptide. Consistent with previous results, the proliferation of OT1 CD8^+^ T cells was also attenuated when co‐cultured with CCL5‐KD Raw264.7 cells (Figure S7, Supporting Information). Moreover, co‐culture assays displayed a decreased percentage of effector CD8^+^ T cells and reduced chemotaxis of CD8^+^ T cells when co‐cultured with CCL5 knockdown macrophages compared to control macrophages (Figure 5G,H).

To gain insight into the molecular mechanisms, GO analysis was carried out on CD8^+^ T cell clusters from the scRNA‐seq data. It revealed enrichment of signaling pathways such as positive regulation of the ERK1 and ERK2 cascade (Figure S8, Supporting Information). This pathway is known to be associated with CCR5 function, as exhibited by Coexpedia (https://www.coexpedia.org/). ERK pathway‐related proteins were then examined in CD8^+^ T cells co‐cultured with RAW264.7‐CCL5‐KD cells. Decreased expression of total proteins of c‐RAF, phosphorylation levels of c‐RAF, P90RSK, and ERK were observed in T cells co‐cultured with RAW264.7‐CCL5‐KD (Figure 5I,J). However, when recombinant CCL5 protein was replenished into the co‐culture cells, the decreased expressions of ERK‐related proteins were rescued (Figure 5I,J). To further validate the interaction between CCL5^hi^ macrophages and CD8^+^ T cells via the CCL5‐CCR5 axis, an anti‐CCR5 antibody Leronlimab, was also employed in co‐culture experiments. Our results demonstrated increased phosphorylation levels of c‐RAF, P90RSK, and ERK in CD8^+^ T cells upon exposure to recombinant CCL5 protein (Figure 5J,K). Notably, the elevations in phosphorylation were effectively abolished when CCR5 was blocked with Leronlimab (Figure 5J,K). Altogether, our results show that CCL5^hi^ macrophages play a crucial role in enhancing cell proliferation, activating CD8^+^ T cells via CCL5‐CCR5 interaction, and increasing the chemotaxis of T cells.

**Figure 6:**
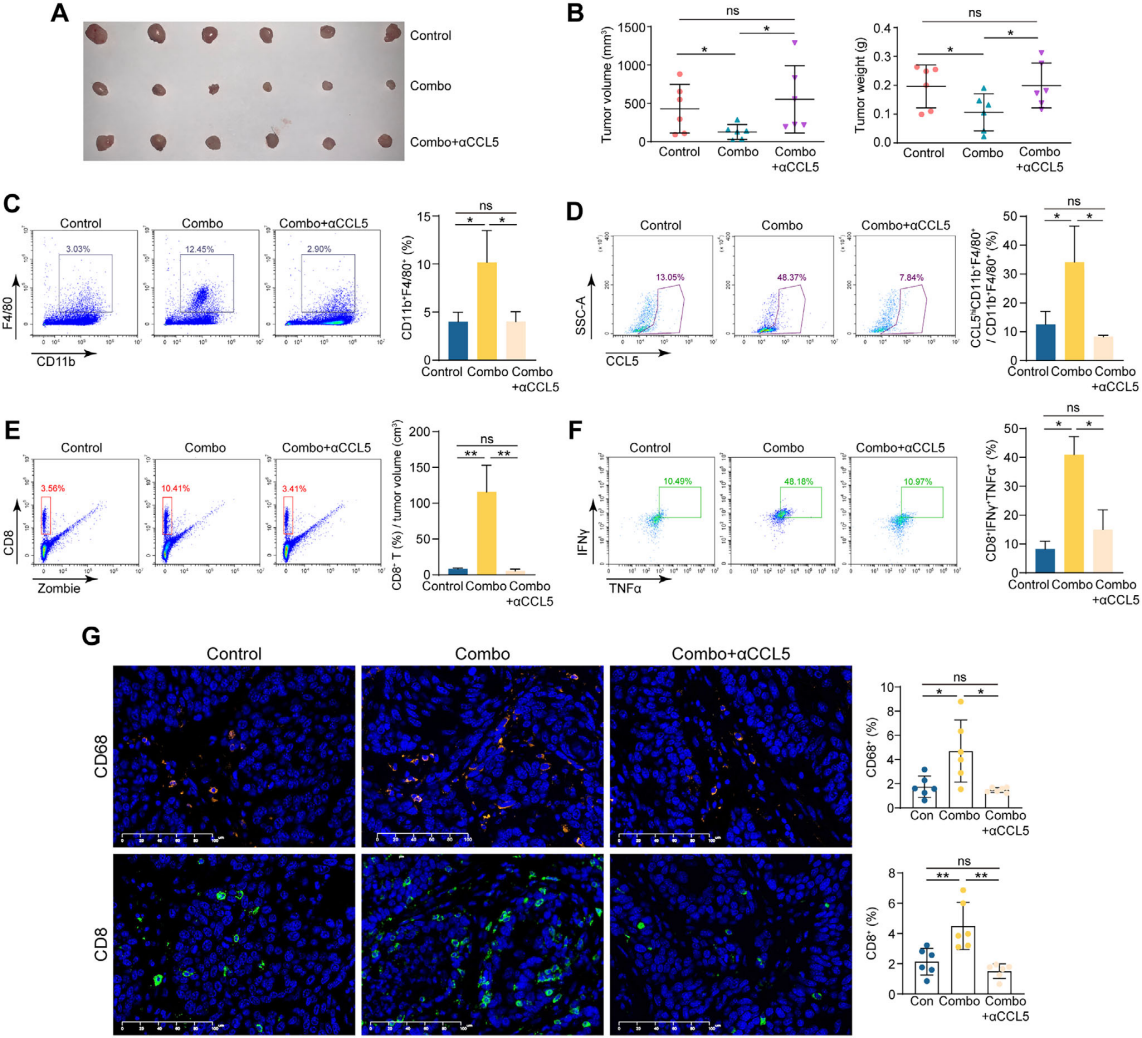
CCL5 antibody weakens the therapy effect of immunochemotherapy in vivo. A) Image of syngeneic tumors formed in C57BL/6J mice that were treated with nab‐PTX+anti‐PD‐1 (combo), nab‐PTX+anti‐PD‐1+anti‐CCL5 (combo+αCCL5), and vehicle control (n = 6 per group). B) Summary of tumor volume and tumor weight of (A) (n = 6 per group; Student's *t*‐test). C,D) Representative flow cytometry plots and summary of CD11b^+^F4/80^+^ cells ratio (C) and the percentage of CCL5^hi^CD11b^+^F4/80^+^ in CD11b^+^F4/80^+^ cells (D) in tumors of combo, combo+αCCL5, and control groups (n = 3 per group; Student's *t*‐test). E) Representative flow cytometry plots and summary of CD8^+^ T cells infiltration (%) normalized by tumor volume (cm^3^) in tumors of combo, combo+αCCL5, and control groups (n = 3 per group; Student's *t*‐test). (F) Representative flow cytometry plots and summary of percentage of CD8^+^IFNγ^+^TNFα^+^ in CD8^+^ T cells in tumors of combo, combo+αCCL5, and control groups (n = 3 per group; Student's *t*‐test). G) Representative images and summary of IF staining of CD68 and CD8 in combo, combo+αCCL5, and control groups (n = 6 per group; Student's *t*‐test). (^*^ *p* < 0.05; ^**^ *p* < 0.01; ns, not significant).

We next tested whether blocking CCL5 could weaken the therapy efficacy of immunochemotherapy. Immune competent mice with tumors were divided into 3 groups: mPD‐1+nab‐PTX (combo), combo+anti‐CCL5, and a blank control group. The in vivo results exhibited that blocking CCL5 significantly compromised the therapeutic effectiveness of immunochemotherapy (**Figure** **6**A,B; Figure S9, Supporting Information). Compared to the control group, the percentage of CD11b^+^F4/80^+^ cells was elevated in the combo group (n = 3; *P*<0.05) (Figure 6C). This increase was abolished by the introduction of an anti‐CCL5 antibody (n = 3; *P*<0.05) (Figure 6C). In addition, the CCL5 expression in macrophages was also examined, and similar results were obtained (Figure 6D). The CCL5 expression was also examined in tumor tissues, and the results demonstrated that the proportion of CCL5^+^ cells was increased in the combo group and the elevation was mitigated in the combo+anti‐CCL5 group (Figure S10, Supporting Information). Moreover, the infiltration of CD8^+^ T cells and the proportion of polyfunctional cells (CD8^+^IFN‐γ^+^TNF‐α^+^) were elevated in the combo group and decreased in the combo+anti‐CCL5 group (Figure 6E,F; Figure S11, Supporting Information). These findings were further supported by IF or IHC assays, which exhibited weakened infiltrations of CD68^+^ and CD8^+^ cells in tumors of the combo+anti‐CCL5 group when compared to the combo group (Figure 6G; Figure S12, Supporting Information).

### 2.5 CCL5 is Transcriptionally Upregulated by Stat1

**Figure 7:**
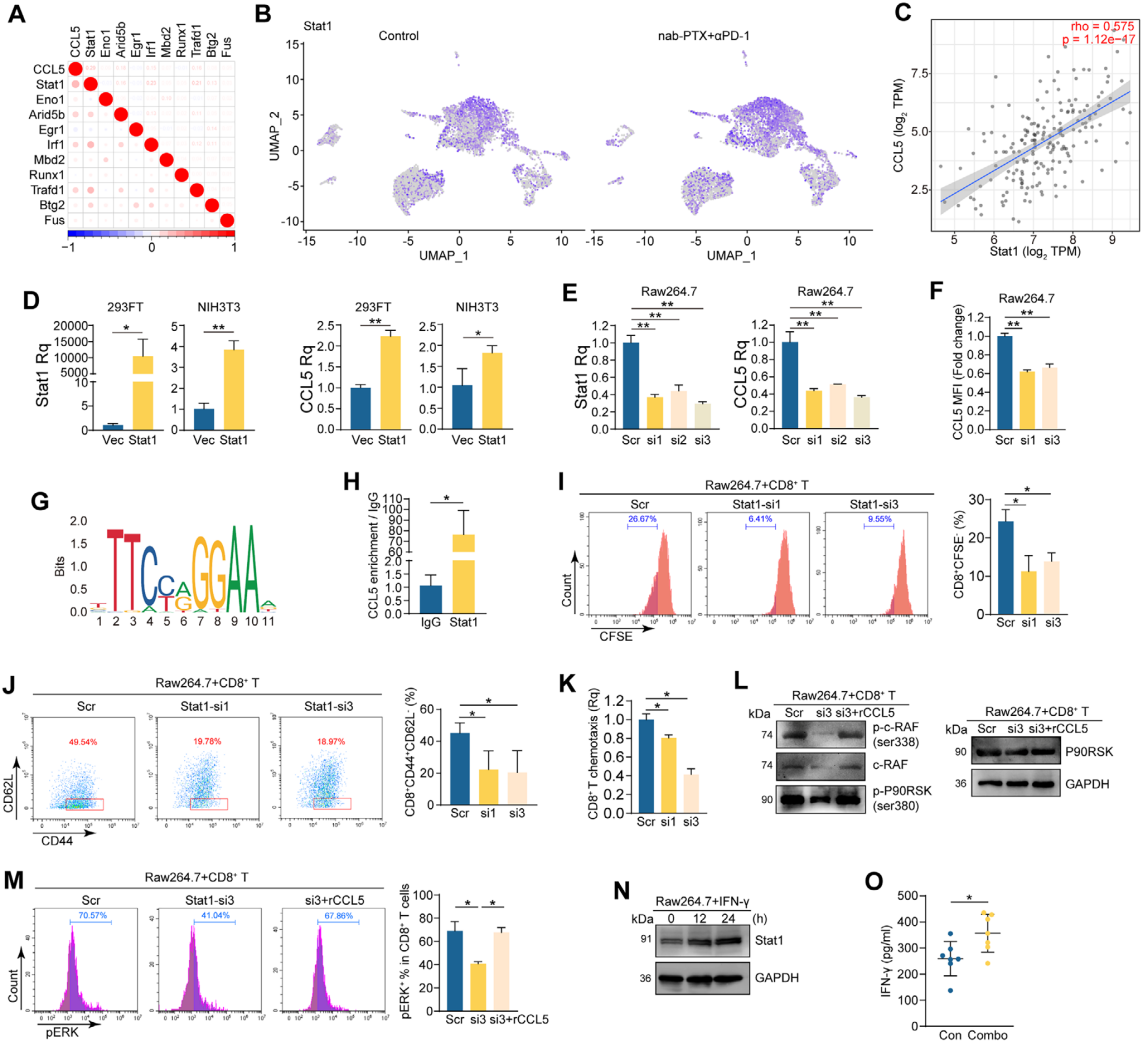
CCL5 is transcriptionally regulated by Stat1 in macrophages. A) Correlation map analyzing the association of CCL5 to transcription factors in the clusters of macrophages and monocytes in the scRNA‐seq data. B) UMAP plot visualization of Stat1 expression in cell clusters from tumors of nab‐PTX+anti‐PD‐1 and control groups. C) Scatter plot showing the correlation between CCL5 and Stat1 in the TCGA‐ESCA dataset (TIMER 2.0 website). D) The RNA levels of Stat1 (*left*) and CCL5 (*right*) in 293FT and NIH3T3 cells overexpressed with Stat1 and control (n = 3 per group; Student's *t*‐test). E) The RNA levels of Stat1 (*left*) and CCL5 (*right*) in RAW264.7 transfected with siRNAs targeting Stat1 or scramble control (n = 3 per group; Student's *t*‐test). F) The relative quantification of MFI of CCL5 in RAW264.7 transfected with siRNAs targeting Stat1 or scramble control (n = 3 per group; Student's *t*‐test). G) The binding sites of Stat1 in the promoter region of CCL5 predicted by JASPER. H) ChIP results of CCL5 enrichment in RAW264.7 cells precipitated with Stat1 antibody and IgG isotype control (n = 3 per group; Student's *t*‐test). I–K) CD8^+^ T cells were co‐cultured with RAW264.7 transfected with siRNAs targeting Stat1 and a scramble control. Representative flow cytometry plots and summary of CD8^+^CFSE^−^/CD8^+^ ratio (I) (n = 3 per group; Student's *t*‐test), CD8^+^CD44^+^CD62L^−^/CD8^+^ ratio (J) (n = 3 per group; Student's *t*‐test), and relative quantification of chemotaxis of CD8^+^ T cells (K) (n = 3 per group; Student's *t*‐test). L,M) CD8^+^ T cells were co‐cultured with RAW264.7‐Stat1‐siRNA or control cells. Then cells were supplemented with recombinant CCL5 protein. Protein levels were determined in CD8^+^ T cells (L). Representative flow cytometry plots and summary of pERK^+^CD8^+^/CD8^+^ ratio (M) (n = 3 per group, Student's *t*‐test). N) The protein level of Stat1 in RAW264.7 treated with IFN‐γ at different time points. GAPDH was a loading control. O) IFN‐γ was determined using ELISA in tumors of nab‐PTX+anti‐PD‐1 (combo) and control groups (n = 7 per group; Student's *t*‐test). (^*^ *p* < 0.05; ^**^ *p*<0.01).

We next investigated the underlying mechanism responsible for CCL5 upregulation in macrophages. ScRNA‐seq data of macrophage and monocyte clusters (Figure 4A) were integrated, and correlation analyses were carried out between transcription factors and CCL5 expression. The correlation coefficient between CCL5 and Stat1 (signal transducer and activator of transcription 1) is 0.29, which is higher than the coefficients for Arid5b (AT‐rich interaction domain 5B) at 0.18, Trafd1 (TRAF‐type zinc finger domain containing 1) at 0.16, or Irf1 (interferon regulatory factor 1) at 0.15 (**Figure** **7**A). Stat1 is also one of the top 50 DEGs of macrophage clusters 1 and 3 when comparing the combo and control groups (Figure 7B). Furthermore, the RNA level of CCL5 is significantly correlated with Stat1 in tumor tissues of the TCGA_ESCA dataset, with a correlation coefficient reaching 0.575 (Figure 7C). To investigate whether Stat1 regulates CCL5, we overexpressed Stat1 in 293FT and NIH3T3 cells and examined the RNA levels of CCL5. The results exhibited that CCL5 was increased in cells overexpressing Stat1 (Figure 7D). Consistently, when Stat1 was silenced in RAW264.7 cells, both the RNA levels and mean fluorescence intensity (MFI) of CCL5 were reduced in Stat1‐KD cells (Figure 7E,F; Figure S13, Supporting Information). Further analysis using JASPAR (a database of transcription factor binding profiles) revealed that Stat1 can bind to the promoter region of CCL5 at the binding site (−1464–−1454 bp) (Figure 7G). Primers were designed based on the binding site, and a ChIP (chromatin immunoprecipitation) assay performed in RAW264.7 cells confirmed that CCL5 was significantly enriched in the anti‐Stat1 pull‐down sample compared with the IgG control sample (*P*<0.05) (Figure 7H). These results indicate that Stat1 is capable of binding to and regulating CCL5 transcriptionally. When CD8^+^ T cells were co‐cultured with Stat1‐KD RAW264.7 or control cells, decreased cell proliferation (Figure 7I), reduced proportion of T_eff_ cells (Figure 7J) and a decrease in chemotaxis ability (Figure 7K) were displayed in CD8^+^ T cells co‐cultured with Stat1‐KD macrophages when compared to those co‐cultured with control macrophages. The phosphorylation levels of c‐RAF, P90RSK, and ERK were reduced in CD8^+^ T cells co‐cultured with Stat1‐KD RAW264.7 cells, which could be rescued by supplementing recombinant CCL5 protein (Figure 7L,M). Tumors were isolated from animals receiving either immunochemotherapy or control, and an mfIHC assay was performed. The proportion of Stat1 nuclear positive CD68^+^ (Stat1^+^CD68^+^) cells in CD68^+^ cells was significantly increased in tumors of immunochemotherapy group compared to that of the control group (n = 6 per group, *P* = 0.03) (Figure S14, Supporting Information). In addition, the ratio of Stat1^+^CD68^+^ cells located near (within 20 µm from) CD8^+^ T cells was increased in tumors of immunochemotherapy group (*P* = 0.04) (Figure S14, Supporting Information).

We next sought to find the mechanism of Stat1 increase in macrophages. Previous studies have suggested that IFN‐γ could induce hyperactivation of Stat1, resulting in the induction of carcinogenesis‐related enzymes in colorectal carcinoma.^[^ ^22^ ^]^ We asked whether IFN‐γ could induce Stat1 in macrophages as well. We treated RAW264.7 cells with IFN‐γ and observed an increase in the protein level of Stat1 (Figure 7N). Furthermore, ELISA results from the animal experiments displayed that the level of IFN‐γ was higher in tumors of immunochemotherapy group than that in the control group (*P* = 0.02, Figure 7O). These findings suggest that IFN‐γ may play a role in inducing Stat1 expression in macrophages, which could potentially contribute to the enhanced efficacy of immunochemotherapy.

### 2.6 Clinical Significance of CCL5^hi^ Macrophages in Patients’ Response to Immunochemotherapy in Multiple Tumors

**Figure 8:**
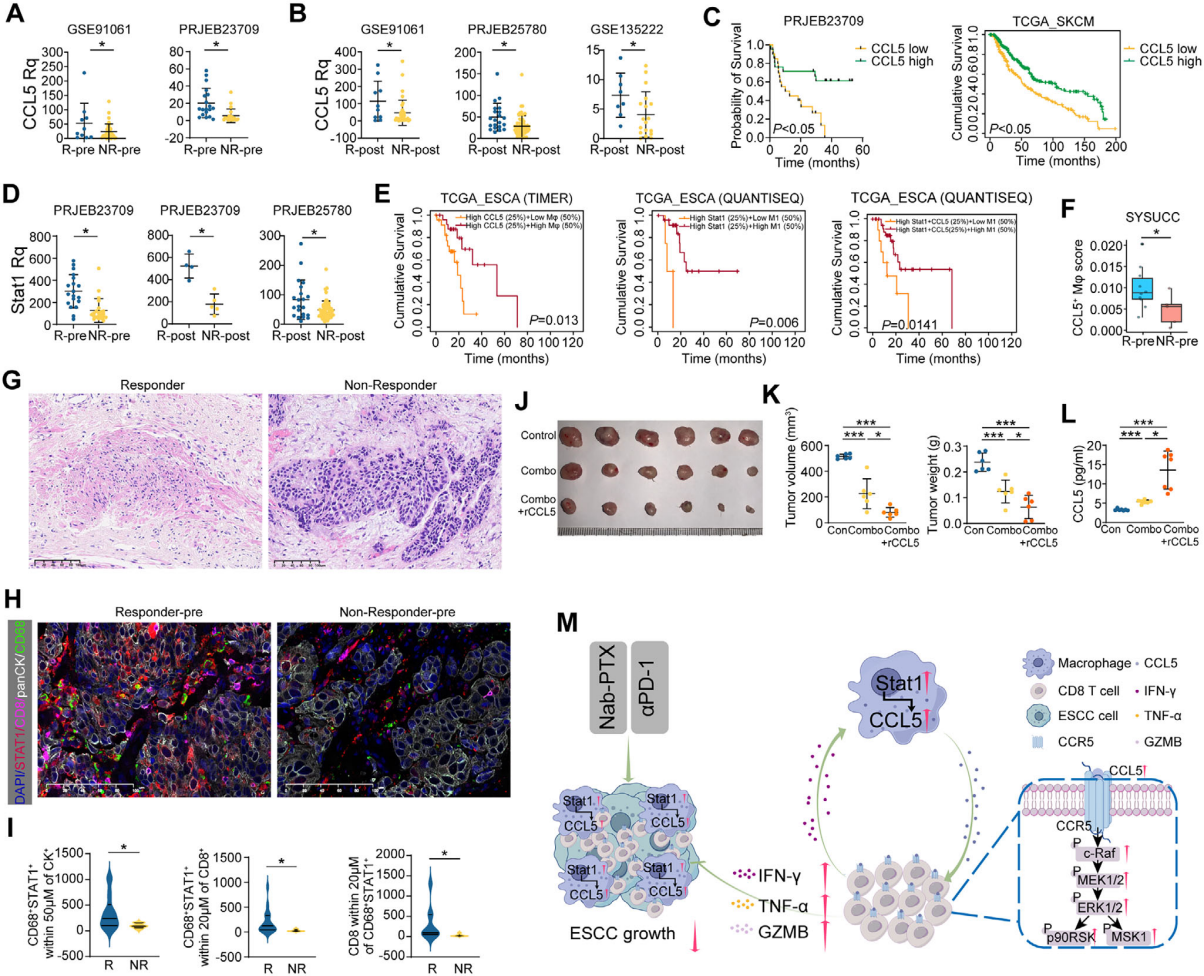
CCL5^hi^ macrophages respond to immunochemotherapy in patients with ESCC. A) Relative quantification of CCL5 in pre‐therapy tumor tissues of responders and non‐responders in GSE91061 (R‐pre = 10; NR‐pre = 39) and PRJEB23709 (R‐pre = 19; NR‐pre = 22) datasets (Wilcoxon signed‐rank test). B) Relative quantification of CCL5 in post‐treatment tumor tissues of responders and non‐responders in GSE91061 (R‐post = 10; NR‐post = 39), PRJEB25780 (R‐post = 21; NR‐post = 57) and GSE135222 datasets (R‐post = 8; NR‐post = 18) (Wilcoxon sign rank test). C) The overall survival of CCL5‐high and CCL5‐low groups of patients in PRJEB23709 and TCGA_SKCM datasets (TIMER 2.0). D) Relative quantification of Stat1 in pre‐therapy or post‐treatment tumor tissues of responders and non‐responders in PRJEB23709 (R‐pre = 19; NR‐pre = 22; R‐post = 4; NR‐post = 5) and PRJEB25780 (R‐post = 21; NR‐post = 57) datasets (Wilcoxon signed‐rank test). E) Kaplan‐Meier plots of the relationship between CCL5, Stat1 combined with macrophage infiltration signatures, and patient overall survival within the TCGA_ESCA dataset (TIMER 2.0). Patients were stratified by CCL5 expression, Stat1 expression or macrophage infiltration status. F) CCL5^+^ macrophages score in pre‐therapy tumor tissues of responders and non‐responders in SYSUCC cohort (RNA‐seq) (R‐pre = 10; NR‐pre = 5; Student's *t*‐test). G) Representative HE images of post‐treatment tumor tissues of responder and non‐responder in SYSUCC cohort. H) Representative mfIHC images of Stat1, CD8, CD68, and panCK staining in pre‐therapy tumor tissues of responder (n = 17) and non‐responder (n = 10) in SYSUCC cohort. I) Summary of spatial distribution of CD68^+^Stat1^+^ macrophages and CD8^+^ T cells in pre‐therapy tumor tissues of responders and non‐responders in SYSUCC cohort (H) (Student's *t*‐test). J,K) Image of tumors formed in C57BL/6J mice that were treated with nab‐PTX+anti‐PD‐1 (combo), nab‐PTX+anti‐PD‐1+recombinant CCL5 protein (combo+rCCL5), and vehicle control (J) (n = 6 per group). Tumor volume and tumor weight were summarized (K) (n = 6 per group; Student's *t*‐test). L) Protein level of CCL5 in tumors of combo, combo+rCCL5, and control groups (n = 7 per group; Student's *t*‐test). M) Proposed model of CCL5^hi^ macrophages respond to immunochemotherapy. (^*^ *p* < 0.05; ^***^ *p* <0.001).

We next downloaded RNA‐seq data from Gene Expression Omnibus (GEO) or China National Center for Bioinformation (CNCB) and analyzed CCL5 in clinical samples from patients who received PD‐1 blockade therapy. The expression of CCL5 is higher in pre‐therapy tissues from responders than that from non‐responders in two melanoma datasets (GSE91061 and PRJEB23709) (**Figure** **8**A). Moreover, the post‐treatment tumor tissues of responders exhibited higher CCL5 expression than those of non‐responders in patients with melanoma (GSE91061), gastric cancer (PRJEB25780), and non‐small cell lung cancer (GSE135222) (Figure 8B). CCL5 high expression level is correlated with better overall survival in patients with melanoma (PRJEB23709 and TCGA_SKCM) (Figure 8C). Correlation analysis revealed that CCL5 correlated significantly with Stat1 in TCGA pan‐cancer data (Figure S15, Supporting Information). Similarly, the RNA level of Stat1 is elevated in pre‐therapy tumor tissues from responders compared with non‐responders in the PRJEB23709 database (Figure 8D). And also, Stat1 is higher in post‐treatment tumor tissues from responders than those from non‐responders in PRJEB23709 and PRJEB25780 (Figure 8D). Next, we incorporated 3 parameters (CCL5, Stat1, and macrophage infiltration) and analyzed overall survival information of patients from the TCGA‐ESCA dataset. The survival results displayed that high CCL5 (top 25%) with high macrophage infiltration (>50%), high Stat1 (top 25%) with high M1‐type infiltration (>50%) or high Stat1 (top 25%) combined with CCL5 (top 25%) and high M1‐type infiltration (>50%) in tumor tissues indicated better overall survival of patients with ESCA (Figure 8E). These findings were further validated in the SYSUCC cohort. RNA‐seq data of SYSUCC showed that the CCL5^+^ macrophage infiltration score was higher in pre‐therapy tumor tissues of responders compared with non‐responders in ESCC patients who received immunochemotherapy (Figure 8F). CD8^+^IFNγ^+^ T cell infiltration scores were also compared based on the bulk RNA‐seq data of SYSUCC cohort. Although the CD8^+^IFN‐γ^+^ T cell infiltration score was higher in pre‐therapy tissues of responders compared to non‐responders, the difference was not statically significant (Figure S16, Supporting Information). Patients with ESCC were categorized into responder or non‐responder groups based on their responses to immunochemotherapy (Figure 8G).

To gain a deeper insight of the spatial distribution of macrophages within tumors, mfIHC was performed on pre‐therapy biopsy specimens using antibodies against CD8, CD68, Stat1, and pan‐CK. Since CCL5 is expressed not only on macrophages but also on some other immune cells (Figure 4C),^[^ ^23^, ^24^ ^]^ CCL5^hi^ macrophages were defined as Stat1 nuclear staining positive macrophages (CD68^+^Stat1^+^). We detected a significantly higher density of CD68^+^Stat1^+^ macrophages within a 50 µm proximity to panCK^+^ tumor cells in responders compared to non‐responders (*P*<0.05) (Figure 8H,I). Moreover, we also noted significant increases in the number of CD68^+^Stat1^+^ macrophages within a 20 µm distance from CD8^+^ T cells, as well as CD8^+^ T cells within a 20 µm distance from CD68^+^Stat1^+^ macrophages, in responders compared to non‐responders (*P*<0.05) (Figure 8H,I). These results indicate that the presence of CD68^+^Stat1^+^ macrophages may be associated with a more favorable response to PD‐1 checkpoint blockade combined with chemotherapy in patients with ESCC.

To further explore the potential clinical application of CCL5, in vivo experiments were carried out, and recombinant CCL5 protein was administered intraperitoneally to mice receiving nab‐PTX and PD‐1 blockade. The results demonstrated that CCL5 administration significantly augmented the therapy effect of immunochemotherapy (Figure 8J,K; Figure S17, Supporting Information). The level of CCL5 in tumors was also examined using ELISA. Results showed that CCL5 in tumor tissues was significantly increased in the combo group and further elevated in the combo+CCL5 group (Figure 8L). Additionally, mfIHC was performed on animal tumor tissues. The results demonstrated that the proportions of CCL5^+^ cells, CCL5^+^ macrophages (CCL5^+^CD68^+^), and Teff cells (CD8^+^CD44^+^CD62L^−^) were significantly elevated in the combo group and further increased in the combo+CCL5 group (Figure S18, Supporting Information).

In summary, our research reveals the existence of a positive feedback loop in ESCC patients receiving immunochemotherapy. Specifically, immunochemotherapy based on PD‐1 blockade increases IFN‐γ, which induces Stat1 elevation in macrophages; followed by CCL5 upregulation via transcriptionally regulated by Stat1; As a result, CD8^+^ T cells are activated by CCL5^hi^ macrophages through CCL5‐CCR5 intercellular communication, and more IFN‐γ is produced (Figure 8M). This positive feedback cycle between IFN‐γ, CCL5^hi^ macrophages, and CD8^+^ T cells was validated through in vivo experiments and clinical specimens. We further propose that recombinant CCL5 protein could enhance the therapeutic efficiency of immunochemotherapy in vivo. Our study offers insights into the mechanism of PD‐1 blockade‐based immunochemotherapy in ESCC and provides a potential therapeutic opportunity to improve the therapeutic effect.

## 3 Discussion

By analyzing bulk RNA‐seq data of the tumor tissues collected before and after NIC (neoadjuvant immunochemotherapy) in patients with ESCC, the infiltration of CD8^+^ T cells and macrophages was assessed in responders and non‐responders of NIC therapy. The immune cell infiltration analyses and mfIHC results suggest that macrophages also play critical roles in the response of ESCC to immunochemotherapy. This was further validated by the observation that therapy efficacy was attenuated by the depletion of macrophages in vivo.

Macrophages are versatile immunocytes that execute a broad spectrum of functions that range from governing tissue homeostasis, defending against pathogens, and helping wound healing.^[^ ^25^ ^]^ As a major cellular component of the tumor microenvironment of many solid tumors, macrophages have been proven to contribute to tumor progression and immunotherapy responses.^[^ ^26^, ^27^ ^]^ Macrophages undergo polarization from M1‐like (classically activated) to M2‐like (alternatively activated) states to maintain the delicate balance between inflammation and regeneration.^[^ ^25^ ^]^ Emerging evidence suggests that tumor progression and metastasis are influenced by dynamic changes of macrophage polarization.^[^ ^28^ ^]^ However, many tumor‐associated macrophages (TAMs) do not fall easily into the simple dichotomy since macrophages are highly heterogeneous in the tumor microenvironment.^[^ ^29^ ^]^ In contrast to the binary M1/M2 distinctions, TAMs have consisted of several particular populations with a complex spectrum of activation states, depending on the tumor types and particular localization.^[^ ^30^ ^]^

Herein, we demonstrated that depletion of macrophages reduced significantly the therapeutic effectiveness of the PD‐1 antibody‐based immunochemotherapy. In addition, we discovered a new population of macrophages with CCL5 high expression in ESCC receiving immunochemotherapy. CCL5, also known as RANTES, functions as a professional chemoattractant that directs the migration of leukocytes into inflammatory lesions in various pathological processes.^[^ ^31^ ^]^ It has been reported that CCL5 performs its biological functions by activating downstream signaling pathways through several cell surface receptors, such as CCR1 (C‐C chemokine motif receptor 1), CCR3, and CCR5.^[^ ^31^, ^32^ ^]^ And also, CCL5 addition directly facilitates M1 polarization and impedes M2 polarization of macrophages through CCL5‐CCR1 and CCL5‐CCR5 mediated activation of MAPK and NF‐κB pathways in drug‐induced liver injury.^[^ ^33^ ^]^

As an important cytokine, CCL5 can induce the infiltration of type I conventional dendritic cells (cDC1) and increase interactions between DC and CD8^+^ T cells.^[^ ^34^ ^]^ Failure to respond to PD‐1 blockade in tumor models could be overcome by intratumoral delivery of CCL5.^[^ ^34^ ^]^ It was also reported that CCL5 overexpression was associated with CD8^+^ T cell infiltration in several types of human solid tumors, and coexpression of CCL5 and CXCL9 has been found to be indicative of immunoreactive tumors with prolonged survival and response to checkpoint blockade.^[^ ^35^ ^]^ An early increase of serum CCL5/CXCL9 levels also identified patients with NK cell‐rich tumors showing favorable responses to anti‐HER2 antibody‐based neoadjuvant treatment in breast cancer patients.^[^ ^23^ ^]^ Reducing CCL5 limited CD8^+^ T cells homing to breast tumors and prevents immune‐mediated destruction of cancer cells.^[^ ^36^ ^]^ However, there are reports indicating that CCL5 plays an opposing role in the tumor microenvironment. For instance, one study suggests that tumor bud‐derived CCL5 can recruit fibroblasts and promote colorectal cancer progression.^[^ ^37^ ^]^ Additionally, gastric cancer cell‐derived CCL5 can recruit TAMs and enhance TNF‐α secretion, thereby increasing lymphatic vessel permeability and facilitating lymphatic metastasis of tumor cells.^[^ ^38^ ^]^ The role of CCL5 remains controversial in ESCC, too. While some studies indicate anti‐tumor effects in ESCC,^[^ ^39^ ^]^ others show that tumor‐derived CCL5 recruits tumor‐associated fibroblasts and promotes tumor growth.^[^ ^40^ ^]^ Single‐cell profiling of ESCC patients undergoing neoadjuvant chemo‐immunotherapy revealed extensive interactions between CCL5 and its receptor CCR5 in various immune cells within pathological complete response (pCR) tumors.^[^ ^41^ ^]^ Herein, we delineate the immune landscape of ESCC tumors in the context of clinical responses to immunochemotherapy. We also analyzed macrophages and identified a subgroup of CCL5^hi^ macrophages in ESCC tumor tissues treated with a combination therapy of PD‐1 blockade and chemotherapy. These CCL5^hi^ macrophages activate CD8^+^ T cells through the CCL5‐CCR5 interaction, leading to the activation of the ERK signaling pathway in CD8^+^ T cells. The ERK signaling pathway plays a crucial role in coordinating T cell metabolism and transcriptional reprogramming, driving T cell differentiation and proliferation.^[^ ^42^ ^]^

It was reported that PD‐1 blockade therapy could induce IFN‐γ.^[^ ^34^ ^]^ In line with these findings, our data indicated that IFN‐γ was increased in mouse tumors treated with immunochemotherapy. Stat1 is an interferon‐induced gene that plays a significant role in the progression of tumors. It is one of the first Stat proteins identified in the IFN signal transduction pathways and has been implicated in suppressing tumor cell proliferation, differentiation, apoptosis, and angiogenesis.^[^ ^43^ ^]^ A recent study has reported that Sta1 activation resulted in CCL2 stimulation, thereby provoking macrophage polarization.^[^ ^44^ ^]^ Similarly, we found Stat1 could be elevated by IFN‐γ treatment in mouse RAW264.7 cells in vitro, and Stat1 transcriptionally upregulated CCL5 in macrophages. Furthermore, supplement with recombinant CCL5 protein could restore the decreased ERK signaling pathway in CD8^+^ T cells co‐cultured with Stat1‐KD macrophages. Our in vivo experiments also indicated that the therapy effect was strengthened by recombinant CCL5 protein in conjunction with immunochemotherapy, potentially providing a safe and effective treatment regimen with translational potential for the effective therapy of ESCC.

Recently, a single‐cell RNA‐seq study was conducted on ESCC treated with neoadjuvant immunotherapy.^[^ ^45^ ^]^ The research identified a population of exhausted SPRY1^+^CD8^+^ T cells exhibiting features of progenitor exhausted T cells.^[^ ^45^ ^]^ CD8^+^Tex‐SPRY1 cells demonstrated the highest number of significant ligand‐receptor interactions with macrophages. Among these interactions, IFNG (interferon gamma) and TNF (tumor necrosis factor) showed strong regulatory potential to induce proinflammatory macrophages.^[^ ^45^ ^]^ We also examined the correlation between SPRY1^+^CD8^+^ T cells and CCL5^hi^ macrophages in our scRNA‐seq data. The interaction strength was relatively weak compared to that between other T cells with CCL5^hi^ macrophages (Figure S19, Supporting Information).

Many clinical trials reported that immune checkpoint blockade therapy plus chemotherapy improved overall survival or progression‐free survival in patients with advanced ESCC.^[^ ^12^, ^13^, ^14^, ^15^, ^16^, ^46^, ^47^ ^]^ However, only some patients benefit from the immunochemotherapy regimen. Therefore, there is an urgent need for molecular signatures or biomarkers that can predict prognosis and response to ICBs therapy. We explored the bulk RNA‐seq data of pre‐therapy ESCC tissues and found that the CCL5^+^ macrophages score was higher in responders compared to non‐responders. In addition, mfIHC results of pre‐therapy tumor tissues also indicated that the abundance of CD68^+^Stat1^+^ cells within pan‐CK^+^ tumor cells or CD8^+^ T cells was associated with a better response to immunochemotherapy in patients with ESCC. Since CD8^+^ T cell infiltration correlates with better survival probability in many cancers, our results suggest that CCL5^hi^ macrophages also play a critical role in ESCC receiving ICBs plus chemotherapy, and CD68^+^Stat1^+^ cell abundance could be a potential biomarker for response to the combined therapy in patients with ESCC.

In conclusion, we depict a comprehensive immune microenvironment in ESCC treated with ICBs plus chemotherapy and identify a specific macrophage subpopulation that may serve as potential predictive markers for the response to immunochemotherapy in patients with ESCC. Our work shed light on the feedback loop involving IFN‐γ, CCL5^hi^ macrophages, and CD8^+^ T cells that is operational in immunochemotherapy for ESCC. Advancement of immunochemotherapy strategies in ESCC will require consideration of other combinations of treatment or immune modulators. Based on our results, supplementing with CCL5 has the potential to enhance the clinical therapeutic effectiveness of the combinatorial immunochemotherapy.

## 4 Experimental Section

### Human Specimens

Locally advanced resectable ESCC patients without distant organ metastases were administered three cycles of chemotherapy (nab‐paclitaxel and capecitabine) and PD‐1 blockade (camrelizumab).^[^ ^15^ ^]^ Response was defined as pathological complete response (pCR) and major pathological response (MPR) (≤ 10% residual viable tumor cells) while non‐response was >10% residual viable tumor cells.^[^ ^15^ ^]^ Tissues were obtained before or after immunochemotherapy using an endoscope or thoracoscopy esophagectomy. The study was approved by the Committees for Ethical Review of Research Involving Human Subjects at Sun Yat‐sen University Cancer Center (B2019‐226‐01 and G2024‐140). This study was conducted in accordance with the ethical guidelines of the Declaration of Helsinki. All informed consent of patients was obtained.

### Cell Lines

Murine ESCC cell line mEC2 was a gift from Dr. Li Fu (Shenzhen University, China).^[^ ^48^ ^]^ Mouse leukemic monocyte/macrophage cell line RAW264.7 was obtained from the Cell Bank of the Chinese, Academy of Sciences (Shanghai, China). 293FT was purchased from Invitrogen (Thermo Fisher Scientific, MA). NIH3T3 was purchased from ATCC (American Type Culture Collection). Cells were validated by STR profiling and tested for no mycoplasma contamination. Cells were grown in DMEM (Gibco BRL, NY) complemented with 10% fetal bovine serum (ExCell, Australia) at 37 °C in 5% CO_2_.

### Bulk RNA Sequencing

Bulk RNA was isolated from tumor tissues using TRIzol (Invitrogen, Carlsbad, CA). Transcriptome sequencing was performed by BGI Tech (BGI Tech, Shenzhen, China). Briefly, high‐quality RNA was obtained after quantification and integrity assessment, and libraries were constructed using MGIEasy RNA Library Prep Kit (BGI Tech, Shenzhen, China). Paired‐end reads (150 bp) were generated on the MGISEQ‐2000 sequencing platform. Clean reads were obtained after low‐quality reads were filtered out.

### Bulk RNA‐seq Data Analyses

Immune cell infiltration was analyzed based on the bulk RNA‐seq data using web resources TIMER 2.0 (http://timer.cistrome.org/). Differentially expressed genes (DEGs) between samples were analyzed by DESeq2. Genes with adjusted *P* value <0.05 were considered as DEGs that were further used for Gene Ontology (GO) enrichment analysis using DAVID (https://david.ncifcrf.gov/). Gene sets that characterize activation, proliferation and chemotaxis of macrophages were downloaded from Molecular Signatures Database (https://www.gsea‐msigdb.org /gsea/msigdb/index.jsp). Gene set enrichment analysis (GSEA) was performed on the RNA‐seq data using GSEA (v4.2.2). The correlations between immune cell infiltration and gene expression were analyzed by Pearson correlation coefficient analysis. For immune cell infiltration abundance analysis, a custom signature matrix file was created from the scRNA‐seq data (for CCL5^hi^ macrophages) or GSE188955 (for CD8^+^IFN‐γ^+^ T cells) by a deconvolution algorithm, CIBERSORT. Then the signature matrix was used to generate the infiltration scores of immune cells using CIBERSORT. Patients with a score = 0 were excluded.

### Public Datasets and Analyses

Transcriptomic data was downloaded (TCGA_ESCA and TCGA_SKCM) generated by The Cancer Genome Atlas (TCGA). RNA‐seq data of PD‐1 blockade, including GSE91061 (melanoma) and GSE135222 (NSCLC), were downloaded from GEO (https://www.ncbi.nlm.nih.gov/), and PRJEB23709 (melanoma) and PRJEB25780 (STAD) from China National Center for Bioinformation (https://ngdc.cncb.ac.cn/bioproject/).

Kaplan–Meier's curves of public datasets (TCGA_SKCM and TCGA_ESCA) were visualized using TIMER 2.0. For PRJEB23709, Kaplan–Meier's curves were generated using GraphPad Prism 9 (La Jolla, CA). For comparison between responders and non‐responders in the four datasets, the Wilcoxon signed‐rank test was performed.

### In Vivo Mouse Models

All animal experiments were approved by the Animal Ethics Committee at Sun Yat‐sen University Cancer Center (L102012022100B). C57BL/6J mice were purchased from Guangdong Medical Animal Center (Guangzhou, China). OT1 mice were purchased from Cavens Model Animal Company Limited (Changzhou, China). Animals were housed under specific pathogen‐free conditions.

### Immunochemotherapy Experiments

mEC2 cells (4×10^6^) suspended in 100 µl DMEM were subcutaneously inoculated into the right flank of 6–8‐week‐old male C57BL/6J mice. When tumors grew to ≈10 mm in length, animals were sacrificed and tumors were removed, chopped into 2 mm^3^ pieces, and implanted subcutaneously into other C57BL/6J mice. One week later, when tumors grew to ≈100 mm^3^, the mice were divided into four groups: control group (normal saline and IgG2a), nab‐PTX group (nab‐PTX and IgG2a), anti‐PD‐1 group (normal saline and anti‐mouse PD‐1), and nab‐PTX+anti‐PD‐1 group (nab‐PTX and anti‐mouse PD‐1). The animals were injected intraperitoneally with drugs or antibodies twice a week for 3 weeks. Reagents used were: anti‐mouse PD‐1 (100 µg mouse^−1^), rat IgG2a control (100 µg mouse^−1^), and nab‐PTX (3 mg kg^−1^). Tumors were measured twice a week, and tumor volume was calculated as volume (mm^3^) = 0.5×L×W^2^ (L: length; W: width). After the experiments, animals were sacrificed and tumors were resected, weighed, and processed.

### Macrophage Depletion Experiments:—Clodronate Liposomes Depletion

Macrophage depletion experiments included pre‐implantation macrophage depletion and post‐implantation macrophage depletion. Clodronate liposomes (100 µl mouse^−1^) and control liposomes were used in the experiments. For the pre‐implantation macrophage depletion assay, animals were divided into 3 groups: control group (normal saline, IgG2a, and control liposomes), nab‐PTX+anti‐mouse PD‐1 group (nab‐PTX, anti‐mouse PD‐1, and control liposomes), nab‐PTX+anti‐mouse PD‐1+clodronate liposomes group (nab‐PTX, anti‐mouse PD‐1, and clodronate liposomes). Clodronate liposomes were injected intraperitoneally into mice 8 times in total: 1 day before tumor implantation, 4 days after tumor implantation, twice a week for 3 weeks from the seventh day after tumor implantation. For the post‐implantation macrophage depletion assay, animals were divided into 2 groups: the nab‐PTX+anti‐mouse PD‐1 group and the nab‐PTX+anti‐mouse PD‐1+clodronate liposomes group. One week after tumor implantation, clodronate liposomes were injected intraperitoneally into mice twice a week for 3 weeks.

### Anti‐CSF1R Depletion

After tumors were established on mice, the mice were divided into 3 groups (n = 6 per group): control group (saline and IgG2a), nab‐PTX+anti‐mouse PD‐1 group, and nab‐PTX+anti‐mouse PD‐1+ anti‐mouse CSF1R group. Anti‐CSF1R (200 µg mouse^−1^) was injected intraperitoneally twice a week for 3 weeks. Nab‐PTX and PD‐1 antibody were utilized as previously described. Rat IgG2a was used as an isotype control.

### Blocking CCL5 Animal Experiments

Animals were divided into 3 groups: control group (normal saline and IgG2a), nab‐PTX+anti‐mouse PD‐1 group (nab‐PTX and anti‐mouse PD‐1), nab‐PTX+ anti‐mouse PD‐1+anti‐mouse CCL5 group (nab‐PTX, anti‐mouse PD‐1, and anti‐mouse CCL5). Anti‐mouse CCL5 (15 µg mouse^−1^) was injected intraperitoneally twice a week for 3 weeks. Rat IgG2a (100 µg mouse^−1^) was used as an isotype control.

### Recombinant Mouse CCL5 Protein Animal Experiments

The assays were replicated twice. In Figure 8J: Animals were divided into 3 groups: control group (normal saline and IgG2a), nab‐PTX+anti‐mouse PD‐1 group (nab‐PTX and anti‐mouse PD‐1), nab‐PTX+anti‐mouse PD‐1+rCCL5 group (nab‐PTX, anti‐mouse PD‐1, and recombinent mouse CCL5 protein) (n = 6 per group). Recombinant mouse CCL5 protein (1 µg mouse^−1^) was injected intraperitoneally twice a week for 3 weeks. In the Figure S17 (Supporting Information): Animals were divided into 2 groups: nab‐PTX+anti‐mouse PD‐1 group, nab‐PTX+anti‐mouse PD‐1+rCCL5 group (n = 7 per group).

### ScRNA‐seq Data Generation and Processing

Mouse tumor tissues were isolated, and CD45^+^ cells were sorted with MojoSort™ Mouse CD45 Nanobeads Kit (Biolegend, San Diego, CA). Single‐cell cellular suspensions were subjected to sequencing in 10K Genomics company (Shanghai, China) as described previously.^[^ ^49^ ^]^ Two single‐cell RNA‐seq libraries were generated from CD45^+^ cells isolated from 3 tumors of each group. Pre‐processing of sorted single‐cell suspensions for library generation was performed using 10× Chromium Controller (10× Genomics, Pleasanton, CA) based on the 10× GEMCode proprietary technology. The initial step consisted in an emulsion where individual cells were isolated into droplets together with gel beads coated with unique primers bearing 10× cell barcodes, unique molecular identifiers (UMI), and poly(dT) sequences. Reverse transcription reactions were engaged to generate barcoded full‐length cDNA, followed by the disruption of emulsions using the recovery agent and cDNA clean‐up with DynaBeads MyOne Silane (10×Genomics, Pleasanton, CA). Sequencing libraries were constructed using the Chromium Next GEM Single Cell 3ʹ Kits v3.1 (10×Genomics, Pleasanton, CA) on a NovaSeq 6000 sequencing system (Illumina, San Diego, CA).

To generate the raw count matrix, sequencing data were aligned on the reference genome GRCm38/84 using CellRanger (version 6.0.1). The matrix was processed in R with the Seurat (v4.3.0.1) package. Only higher‐quality cells (mitochondrial content < 10%, number of detected genes > 200) and genes (cell number > 3) were further analyzed. Potential double cells were filtered out with the Scrublet package.^[^ ^50^ ^]^ LogNormalize was used to normalize and scale the filtered expression matrix. FindVariableFeatures was applied to calculating variable features for each sample, and principal component analysis (PCA) was performed based on the 2000 most variable genes (RunPCA). Clustering was processed using Uniform Manifold Approximation and Projection (UMAP) dimension reduction with a resolution of 1.3. The FindAllMarkers function was applied to obtain DEGs and characterize clusters. Genes with a significant difference (*P* <0.05, logFold‐Change ≥ 0.25) were further analyzed. Cellular communication analysis was performed based on a curated public dataset of 2,557 known receptor‐ligand interaction pairs.^[^ ^51^ ^]^ To identify the transcription factor/cell surface marker most closely related to CCL5, a Pearson correlation coefficient was performed in monocyte and macrophage clusters.

### Immunofluorescence (IF)

The paraffin‐embedded tissue sections were deparaffinized by xylene, rehydrated with a series of graded ethanol. After antigen retrieval with EDTA buffer, tissue sections were incubated with primary antibody at 4 °C overnight and then with fluorescent secondary antibody at 37 °C for 30 min. Nuclei were counterstained with DAPI (Abcam, Cambridge, UK). The slides were scanned by the KF‐PRO‐020 system (KFBIO, Ningbo, China)

### Immunohistochemistry (IHC)

The paraffin‐embedded tissue sections were deparaffinized by xylene, rehydrated with a series of graded ethanol, then blocked with 3% hydrogen peroxide solution. After antigen retrieval with EDTA buffer, tissue sections were incubated with primary antibody at 4 °C overnight and then with horseradish peroxidase‐conjugated secondary antibody at 37 °C for 30 min. The staining results were visualized using DAB (ZSGB‐BIO, Beijing, China). The slides were scanned by the KF‐PRO‐020 system (KFBIO, Ningbo, China).

### Multiplex Fluorescence IHC (mfIHC)

The paraffin‐embedded tumor tissues were analyzed by mfIHC staining using the Opal 5‐color Manual IHC kit (PANOVE, Beijing, China) according to the manufacturer's protocol. Briefly, tissue sections were deparaffinized, rehydrated, and blocked prior to antigen recovery in EDTA buffer. Afterwards, the sections underwent 4 sequential cycles of staining procedures. Each cycle included antigen recovery, blocking, binding of primary antibody, the corresponding secondary antibody, and incubation with paired Opal tyramide signal amplification reagent. After the 4‐cycle staining procedures, the nuclei were counterstained with DAPI (Abcam, Cambridge, UK). The slides were scanned by the KF‐PRO‐020 system (KFBIO, Ningbo, China). Staining quantification and spatial analysis were carried out using HALO 3.3 image analysis software (Indica Labs, Albuquerque, NM). Antibodies were used sequentially: Stat1, CD8, CD68 and panCK; or CCL5, CD68; CD8, CD44, CD62L.

### Plasmid, siRNAs, and Transfection

Plasmid HA‐PCDH‐mStat1 was purchased from YOUBIO (Changsha, China). SiRNAs targeting CCL5 or and Stat1 were ordered from RIBOBIO (Guangzhou, China). Cells were transfected with plasmid or siRNAs using HilyMax transfection reagent (Dojindo, Japan) according to the standard protocol. Briefly, Raw264.7 cells were seeded in a 24‐well plate at a confluence of 30%. After 24 h, the medium was substituted with Opti‐MEM (Thermo Fisher Scientific, MA). Subsequently, either 2 µg DNA or 40 nM siRNA was mixed with HilyMax transfection reagent to form the transfection complex. Fifteen minutes later, the cells were transfected with the mixture and cultured for 36 h.

### Flow Cytometry

Mouse tumor tissues were harvested, digested with collagenase IV and DNase I, and lysed with blood cell lysis buffer. Cell suspensions were filtered on a 70 µm cell‐strainer (BD Biosciences, San Jose, CA) and stained with Zombie Aqua™ kit (Biolegend, San Diego, CA). For cell surface antibody staining (CD8, CD11b, F4/80, CD86, CD206, and Ly6a), cells were washed and stained with fluorophore‐conjugated antibodies in the presence of Fc‐receptors blocking reagent anti‐mCD16/32. For intracellular staining (CCL5, TNF‐α, and IFN‐γ), cell suspensions were subsequently washed with buffer and submitted to intracellular staining after fixation and permeabilization according to the standard protocols. Regarding the detection of TNF‐α and IFN‐γ, cells were first treated with Brefeldin A (Biolegend, San Diego, CA) to block transport processes before Fc‐receptor blocking. Data acquisition was performed using a CytoFLEX LX system (Beckman Coulter, Brea, CA) for cell analysis or a MoFlo Astrios system (Beckman Coulter, Brea, CA) for cell sorting.

### Cell Co‐Culture Assay

Mouse tumors were harvested, digested using collagenase IV (1.5 mg ml^−1^) and DNase I (0.5 U ml^−1^). Subsequently, the cells were lysed with blood cell lysis buffer. The cell suspension was filtered through a 70 µm cell‐strainer (BD Biosciences, San Jose, CA). Fc‐receptors were blocked using anti‐mCD16/32. Following this, the suspension was stained with Zombie Aqua™ kit (Biolegend, San Diego, CA). Then the cells were stained for CD11b, F4/80, and Ly6a. CD11b^+^F4/80^+^Ly6a^+^ cells were sorted using the MoFlo Astrios system (Beckman Coulter, Brea, CA).

CD8^+^ T cells were isolated from the spleens of healthy C57BL/6J or OT1 mice using MojoSort™ Mouse CD8 T Cell Isolation Kit (Biolegend, San Diego, CA). CD11b^+^F4/80^+^Ly6a^+^ macrophages (1×10^4^) isolated from mouse tumors were co‐cultured with CD8^+^ T cells (5×10^4^) in 96‐well plates. The plates were incubated with anti‐mouse CD3ε antibody (0.5 µg ml^−1^) for 2 h at 37 C, followed by washing with PBS. The cells were co‐cultured in complete RPMI1640 supplemented with anti‐mouse CD28 antibody (0.5 µg ml^−1^). Two days later, the proportion of lineage CD8^+^CD44^+^CD62L^−^ T cells was examined. Similarly, RAW264.7 (1×10^5^) cells were co‐cultured with CD8^+^ T cells (5×10^5^) in the presence of CD3ε and CD28 antibodies in 24‐well plates, and assays were performed after 3 days of co‐culture. In the co‐culture experiments, recombinant CCL5 protein (0.5 µg ml^−1^) or leronlimab (40 ug ml^−1^) was introduced to the co‐cultured cells.

### T Cell Proliferation Assay and T Cell Activation Assay

RAW264.7 cells were co‐cultured with CFSE (carboxyfluorescein succinimidyl ester, Absin, Shanghai, China) labeled CD8^+^ T cells as described above. Three days later, the fluorescence intensity of CFSE was examined using flow cytometry. In the co‐culture system of RAW264.7‐CCL5‐KD cells and CFSE‐labeled CD8⁺ T cells, recombinant IL‐2 protein was added at a concentration of 50 ng ml^−1^. For the proliferation assay of CD8⁺ T cells derived from OT1 mice, the culture medium was supplemented with 100 nM SIINFEKL OVA peptide and 50 ng ml^−1^ IL‐2.

After cell co‐culture, T cells were stained for CD8, CD44, and CD62L to analyze cell activation status using flow cytometry.

### T Cell Chemotaxis Assay

CD8^+^ T cells were plated in the upper chamber with 5 µm‐pore (BD Biosciences, San Jose, CA) in complete RPMI1640 medium supplemented with anti‐CD3ε (0.5 µg ml^−1^) and anti‐CD28 (0.5 µg ml^−1^). RAW264.7 cells were plated in the lower chamber. After 1 day of co‐culture, cell chemotaxis was examined by counting CD8^+^ T cells in the lower chamber using 123count eBeads™ Counting Bead (Thermo Fisher, Waltham, MA).

### Western Blotting

Co‐cultured CD8^+^ T cells were collected using MojoSort™ Mouse CD8 T Isolation Kit (Biolegend, San Diego, CA). Cells were lysed using RIPA buffer. Western blotting was carried out according to the standard protocol. The results were captured using the Gel Imaging System (Bio‐Rad, Hercules, CA).

For Stat1 detection, RAW264.7 cells were treated with IFN‐γ (20 ng ml^−1^) for different time points (0 h, 12 h, and 24 h). Cells were then harvested, and western blotting was performed.

### Quantitative Real‐Time PCR (qRT‐PCR) Assay

Total RNA from cells was harvested using RNA‐Quick Purification Kit (ES Science, Shanghai, China) and reverse transcribed with PrimeScript Reverse Transcription Kit (Takara, Japan). QRT‐PCR assay was carried out using FastStart Universal SYBR Green Master (Roche, Basel, Switzerland) on LightCycler 480 system (Roche, Basel, Switzerland). The sequences of primers were listed in Supporting Information.

### Chromatin Immunoprecipitation (ChIP)

ChIP assay was performed using the Simple ChIP Enzymatic ChIP Kit (Cell Signaling Technology, Boston, MA). RAW264.7 (2×10^7^) cells were cross‐linked with 1% formaldehyde for 10 min at room temperature. Chromatin digestion was performed with 1.0 µl micrococcal nuclease, and cells were sonicated to break the nuclear membrane. DNA‐protein complexes were immunoprecipitated with Stat1 antibody or rabbit IgG isotype control. Primers were designed based on the potential binding sites of Stat1 on the CCL5 promoter predicted by JASPAR (https://jaspar.elixir.no/). Results were quantified using SYBR Green on the LightCycler 480 system (Roche, Basel, Switzerland). The enrichment of specific genomic regions for the anti‐Stat1 group was calculated relative to the IgG control group by the 2^−ΔΔCt^ method.

### Enzyme‐Linked Immunosorbent Assay (ELISA) Assay

ELISA kits for CCL5 or IFN‐γ were purchased from Abclonal (Wuhan, China). Mouse tumor tissues (100 mg) were minced, sonicated, and centrifuged at 5000 g for 10 min at 4°C. The supernatant was collected, and an ELISA was performed according to the manufacturer's protocol. The optical density was determined at 450 nm using a microplate reader (BioTek, Winooski, VT). For CCL5 detection, samples of combo and combo+rCCL5 groups were from a recombinant mCCL5 animal experiment (Figure S17, Supporting Information).

### Statistical Analyses

Data was expressed as mean±S.D. Statistical analyses were performed using SPSS 23.0 (SPSS, Inc., Chicago, IL) or GraphPad Prism 9.0 (La Jolla, CA). A two‐tailed Student's *t*‐test was employed to evaluate the differences between two groups. *P*< 0.05 was considered statistically significant. Statistical analyses were indicated in figure captions.

## Conflict of Interest

The authors declare no conflict of interest.

## Author contributions

Y.M. and X.S. contributed equally to this work. Y.M. performed the experiments, analyzed data, and wrote the draft; T.Z. and C.Z. performed the experiments; Z.X. and J.Z. analyzed data; X.S., G.Y., C.Z., and X.Z. provided the clinical samples and information. X.Y.G. provided funding support. Y.L. designed and supervised the study, provided funding support, and wrote the manuscript. All authors read and approved the manuscript.

## Supporting information



Supporting Information

Supporting Information

## Data Availability

The data that support the findings of this study are available from the corresponding author upon reasonable request.
