# Supplementary material for: CCL5hi Macrophages Interact with CD8+ T Cells and Potentiate Responsiveness to PD‐1 Blockade Plus Chemotherapy in Esophageal Squamous Cell Carcinoma
Source: Adv Sci (Weinh). 2025 Sep 25;12(45):e09187. doi: 10.1002/advs.202409187 (PMC12677597; doi:10.1002/advs.202409187)
Supplement: Supplementary file 1 — Supporting Information [file ADVS-12-e09187-s002.docx]

**Supplementary materials:**

**Sequences of primers:**

| **Name** | **Sequences** |
| --- | --- |
| CCL5(mouse) | Forward: 5' GACACCACTCCCTGCTGCTTTG 3'  Reverse: 5' CTCTGGGTTGGCACACACTTGG 3' |
| Stat1 (mouse, human) | Forward: 5' TGCTGTGCCTCTGGAATGATGG 3'  Reverse: 5' CCTGGCTGCTGGTCCTTGAG 3' |
| CCL5 (human) | Forward: 5' CAGCAGTCGTCCACAGGTCAAG 3'  Reverse: 5' TTTCTTCTCTGGGTTGGCACACAC 3' |
| β-actin (human) | Forward: 5' CACCATTGGCAATGAGCGGTTC 3'  Reverse: 5' AGGTCTTTGCGGATGTCCACGT 3' |
| β-actin (mouse) | Forward: 5' GGCTGTATTCCCCTCCATCG 3'  Reverse: 5' CCAGTTGGTAACAATGCCATGT 3' |
| CCL5-ChIP | Forward: 5'-TCAGATCACATGTCACACACTAA-3'  Reverse: 5'-TGTTGTAAGGAATTTGCCAGGT-3' |

**Key resources:**

| **Reagent or Resource** | | **Source** | | **Identifier** | | | |  |  |
| --- | --- | --- | --- | --- | --- | --- | --- | --- | --- |
| **Antibodies or reagents** | |  | |  | | | |  |  |
| anti-CD8 | | | ZSGB-BIO | | | | ZA-0508 | | |
| anti-CD68 (human) | | | ZSGB-BIO | | | | ZM-0060 | | |
| anti-panCK | | | MXB Biotechnologles | | | | RAB-0050 | | |
| anti-CCL5 | | | CST | | | | 36467S | | |
| anti-CD44 | | | Abclonal | | | | A0340 | | |
| anti-CD62L | | | Abclonal | | | | A1622 | | |
| anti-CD8α | | | CST | | | | 98941S | | |
| anti-CD68 (mouse) | | | Abcam | | | | ab955 | | |
| anti-PD-1 | | | BioXCell | | | | BE0273 | | |
| Rat IgG2a, κ | | | BioXCell | | | | BE0089 | | |
| anti-CCL5 | | | Biolegend | | | | 947004 | | |
| anti-CSF1R | | | Selleck | | | | A2159 | | |
| IL-2 | | | Abclonal | | | | RP01384 | | |
| Opti-MEM | | | Thermo Fisher | | | | 31985070 | | |
| Leronlimab | | | Targetmol | | | | T77059 | | |
| rCCL5 | | | Abclonal | | | | RP02719 | | |
| anti-c-RAF | | | CST | | | | 9154S | | |
| anti-p-c-RAF (ser338) | | | CST | | | | 9427S | | |
| anti-p-P90RSK (ser380) | | | CST | | | | 11989S | | |
| anti-P90RSK | | | CST | | | | 9595S | | |
| anti-STAT1 | | | CST | | | | 14994S | | |
| anti-GAPDH | | | Proteintech | | | | 10494-1-AP | | |
| collagenase IV | | | Sigma | | | | C5138 | | |
| DNase I | | | Thermo Fisher | | | | EN0521 | | |
| blood cell lysis buffer | | | KeyGEN BioTECH | | | | KGB5201 | | |
| FITC anti-mouse CD11b | | | Biolegend | | | | 101206 | | |
| APC anti-mouse F4/80 | | | Biolegend | | | | 123116 | | |
| Pacific Blue (PB) anti-mouse CD8a | | | Biolegend | | | | 100725 | | |
| PE/Cyanine7 anti-mouse CD86 | | | Biolegend | | | | 105041 | | |
| FITC anti-mouse CD44 | | | Biolegend | | | | 103022 | | |
| APC anti-mouse CD62L | | | Biolegend | | | | 104411 | | |
| PE anti-mouse Ly6a | | | Biolegend | | | | 108107 | | |
| Brilliant Violet (BV) 421 anti-mouse CD206 | | | Biolegend | | | | 141717 | | |
| PE anti-mouse CCL5 | | | Biolegend | | | | 149104 | | |
| PE/Cyanine7 anti-mouse CCL5 | | | Biolegend | | | | 149105 | | |
| APC/Cyanine7 anti-mouse TNF-α | | | Biolegend | | | | 506343 | | |
| PE anti-mouse IFN-γ | | | Biolegend | | | | 505807 | | |
| APC anti-mouse pERK | | | Biolegend | | | | 369521 | | |
| purified anti-mouse CD3ε antibody | | | Biolegend | | | | 100339 | | |
| purified anti-mouse CD28 antibody | | | Biolegend | | | | 102115 | | |
| HRP-labeled secondary antibody | | | ZSGB-BIO | | | | PV-6000 | | |
| Alexa Fluor™ 488 Goat anti-Rabbit IgG (H+L) | | | Thermo Fisher | | | | A11008 | | |
| Alexa Fluor™ 546 Goat anti-Mouse IgG (H+L) | | | Thermo Fisher | | | | A11030 | | |
| MojoSort™ Mouse CD45 Nanobeads Kit | | | Biolegend | | | | 480027 | | |
| MojoSort™ Mouse CD8 T Cell Isolation Kit | | | Biolegend | | | | 480008 | | |
| CFSE | | | Absin | | | | abs9106 | | |
| Permeabilization buffer | | | Thermo Fisher | | | | 00-8333-56 | | |
| Fixation buffer | | | Thermo Fisher | | | | 00-8222-49 | | |
| 123count eBeads™ Counting Bead | | | Thermo Fisher | | | | 01-1234-42 | | |
| Mouse CCL5 Elisa Kit | | | Abclonal | | | | RK00167 | | |
| Mouse IFN-γ Elisa Kit | | | Abclonal | | | | RK00019 | | |
| Simple ChIP Enzymatic ChIP Kit | | | CST | | | | 9003 | | |
| PANO 5-plex IHC Kit | | | Panovue | | | | 10293100100 | | |
| DAB | | | ZSGB-BIO | | | | ZLI-9018 | | |
| DAPI | | | Abcam | | | | 104139 | | |
| HilyMax transfection reagent | | | Dojindo | | | | H357 | | |
| RNA-Quick Purification Kit | | | ES Science | | | | RN001 | | |
| PrimeScript Reverse Transcription Kit | | | Takara | | | | RR047A | | |
|  | |  | |  | | | |  |  |
| **Animal** | |  | |  | | | |  |  |
| C57BL/6J | | Guangdong Medical Animal Center | | | | | |  |  |
| OT1 | | Changzhou Cavens Model Animal Company Limited | | | | | |  |  |
| **Cell lines** | |  | | |  | | |  |  |
| Raw264.7 | | Cell Bank of Chinese, Academy of Sciences | | | TCM13 | | |  |  |
| mEC2 | | A kind gift from Dr. Li Fu (Shenzhen University, China) | | | | | |  |  |
| 293FT | | Invitrogen | | | R70007 | | |  |  |
| NIH3T3 | | ATCC | | | bio-68106 | | |  |  |
|  |  | | |  | | | |  |  |
| **Public databases** |  | | |  | | | |  |  |
| TCGA_ESCA | | TCGA | | https://portal.gdc.cancer.gov/v1 | | | |  |  |
| TCGA_SKCM | | TCGA | | https://portal.gdc.cancer.gov/v1 | | | |  |  |
| GSE91061 | | GEO | | https://www.ncbi.nlm.nih.gov/ | | | |  |  |
| GSE135222 | | GEO | | https://www.ncbi.nlm.nih.gov/ | | | |  |  |
| PRJEB23709 | | CNCB | | https://ngdc.cncb.ac.cn/bioproject/ | | | |  |  |
| PRJEB25780 | | CNCB | | https://ngdc.cncb.ac.cn/bioproject/ | | | |  |  |
|  | |  | |  | | | |  |  |
| **Software and algorithms** | |  | |  | | | |  |  |
| R | | R Foundation for Statistical Computing (https://www.r-project.org/) | | | | | | |  |
| Cell Ranger | | 10X Genomics (http://10xgenomics.com/) | | | | | | |  |
| Scrublet package | | https://github.com/swolock/scrublet | | | | | | |  |
| Seurat | | https://satijalab.org/seurat/ | | | | | | |  |
| SPSS | | SPSS, Inc., Chicago, IL | | | |  | | |  |
| GraphPad Prism | | GraphPad (La Jolla, CA) | | | |  | | |  |

**Supplementary tables:**

**Supplementary table 1: Cell clusters and marker genes in scRNA-seq data.**

| **Cell type** | **Marker gene** | **Control** | | **nab-PTX+αPD-1** | |
| --- | --- | --- | --- | --- | --- |
|  |  | **Proportion** | **Number** | **Proportion** | **Number** |
| B cell | Cd79a, Cd79b, Mzb1 | 0.32 | 25 | 0.72 | 58 |
| DC | Cst3 | 2.72 | 211 | 2.73 | 220 |
| Endothelial | Pecam1, Vwf | 1.28 | 99 | 1.18 | 95 |
| Epithelial | Epcam, Krt5 | 0.33 | 26 | 0.43 | 35 |
| Fibroblast | Col1a1, Col1a2, Dcn | 6.48 | 503 | 1.81 | 146 |
| Macrophage 1 | C1qa, C1qb, C1qc | 8.82 | 685 | 17.38 | 1402 |
| Macrophage 2 |  | 16.14 | 1253 | 10.60 | 855 |
| Macrophage 3 |  | 0.73 | 57 | 7.66 | 618 |
| Macrophage 4 |  | 9.71 | 754 | 5.35 | 432 |
| Macrophage 5 |  | 4.50 | 349 | 9.02 | 728 |
| Monocyte | Vcan, Lyz2 | 5.91 | 459 | 3.14 | 253 |
| Neutrophil | S100a8, S100a9 | 32.71 | 2539 | 21.29 | 1718 |
| CD8^+^ Effector Tcell | Cd3d, Cd8a, Ptprc | 3.23 | 251 | 6.14 | 495 |
| CD8^+^ Memory Tcell | Cd3d, Cd8a, Ptprc^-^ | 0.79 | 61 | 2.89 | 233 |
| NK Tcell | Cd3d, Ets1 | 1.83 | 142 | 3.88 | 313 |
| NK | Cd3d^-^, Nkg7 | 1.43 | 111 | 2.69 | 217 |
| Treg | Foxp3 | 1.46 | 113 | 2.85 | 230 |
| Th17 T cell | Il17a | 1.61 | 125 | 0.25 | 20 |

**Supplementary table 2. Interactions of ligand-receptor between CCL5^hi^ macrophages and CD8^+^ effector T cells in Combo group.**

| **source** | **target** | **ligand** | **receptor** | **probability** |
| --- | --- | --- | --- | --- |
| Ccl5^hi^ macrophages | CD8^+^ Effector T cells | CCL5 | CCR5 | 0.018176809* |
| Ccl5^hi^ macrophages | CD8^+^ Effector T cells | THBS1 | CD47 | 0.014245191** |
| Ccl5^hi^ macrophages | CD8^+^ Effector T cells | FN1 | ITGA4_ITGB7 | 0.002838676** |
| Ccl5^hi^ macrophages | CD8^+^ Effector T cells | FN1 | ITGAV_ITGB1 | 0.002809555** |
| Ccl5^hi^ macrophages | CD8^+^ Effector T cells | CD80 | CTLA4 | 0.002769051** |
| Ccl5^hi^ macrophages | CD8^+^ Effector T cells | CD80 | CD28 | 0.002654486** |

Note: *, *P*<0.05; **, *P*<0.01.

**Supplementary figure legends:**


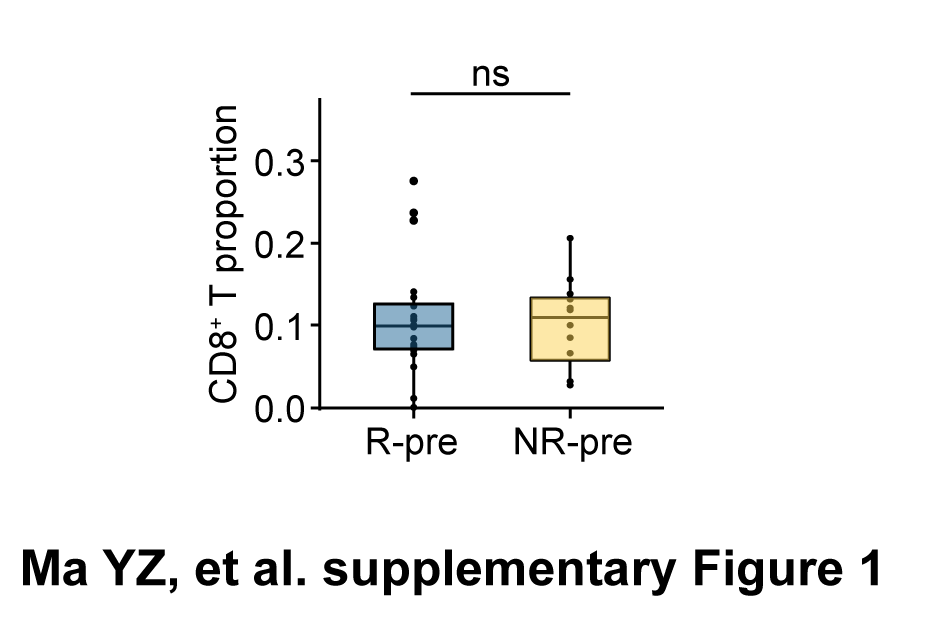


**Supplementary figure 1: The proportion of CD8^+^ T cells in the pre-therapy tissues of responders and non-responders.**

The proportion of CD8^+^ T cells in the pre-therapy tissues of responders (n=20) and non-responders (n=11) (Student’s *t*-test). (ns, not significant)


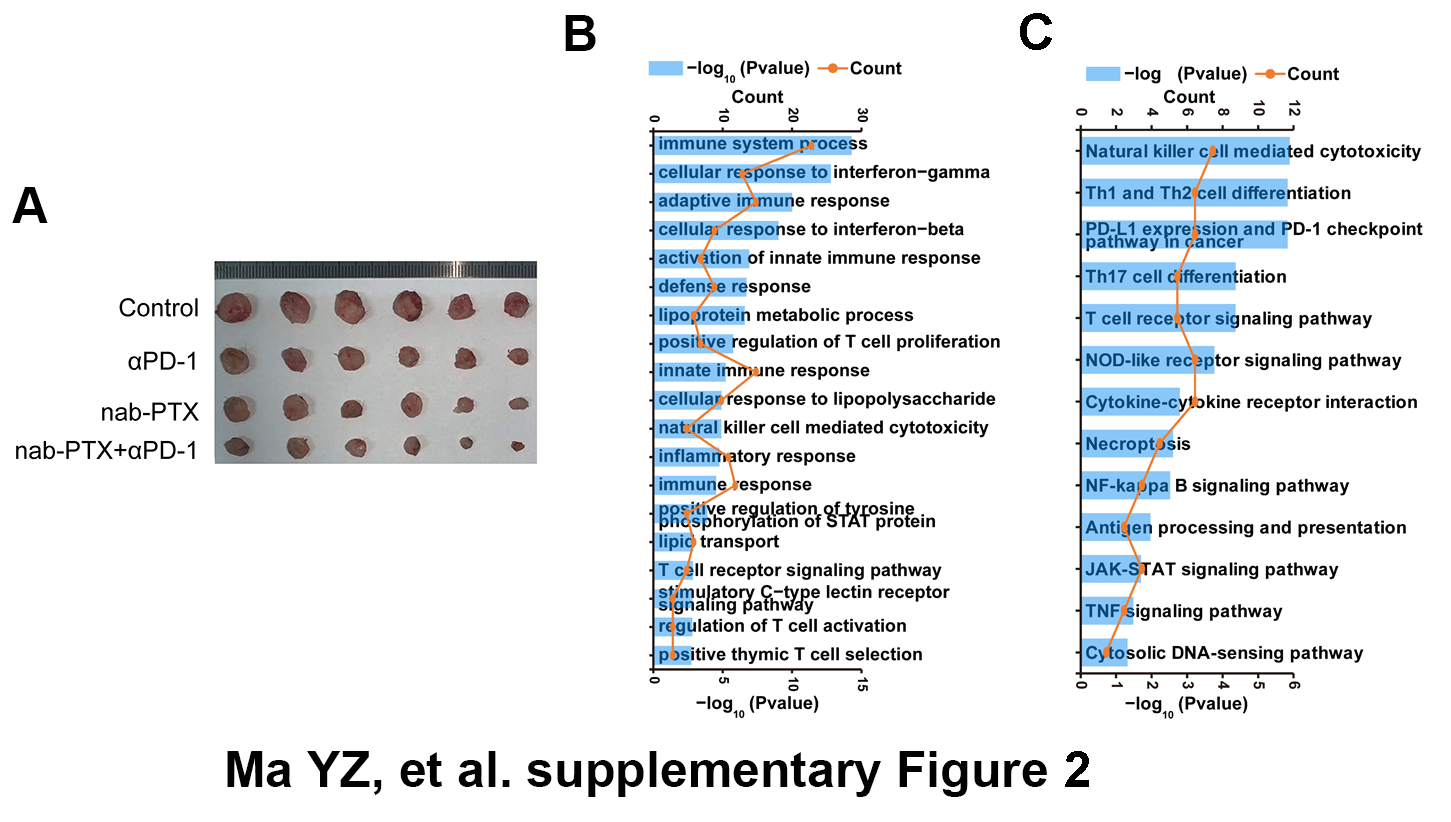


**Supplementary figure 2: Analyses of DEGs between the immunochemotherapy and control groups.**

**A)** Figure 2A with scale. **B-C**) Biological process analysis (**B**) and KEGG analysis results (**C**) of the top 150 DEGs between the immunochemotherapy (nab-PTX+anti-PD-1) and vehicle control groups.


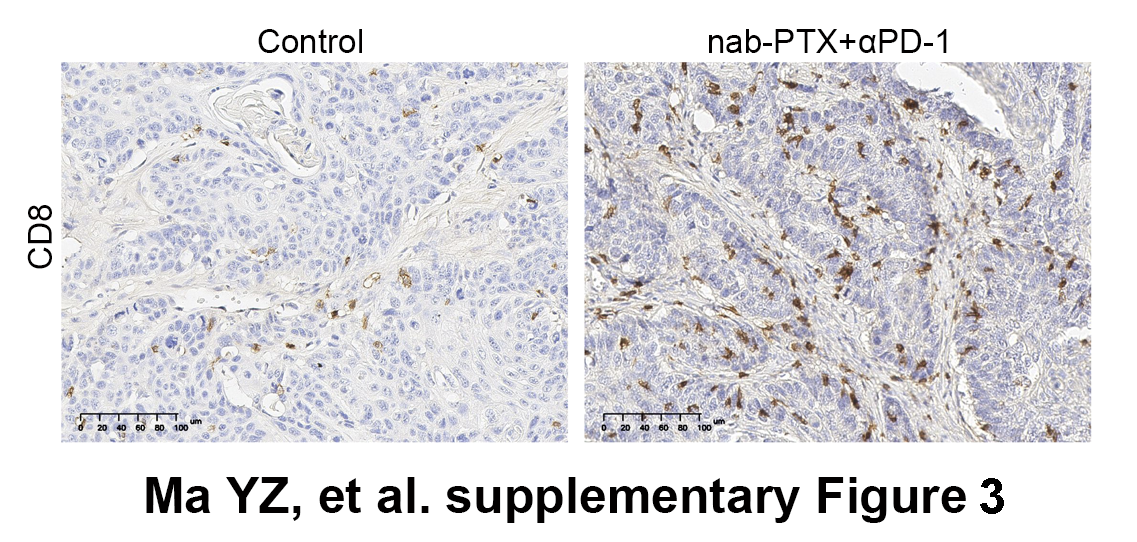


**Supplementary figure 3: IHC staining of CD8 in tumors of the immunochemotherapy and control groups.**

Representative images of CD8 staining in tumors of the immunochemotherapy (nab-PTX+anti-PD-1) and vehicle control groups.


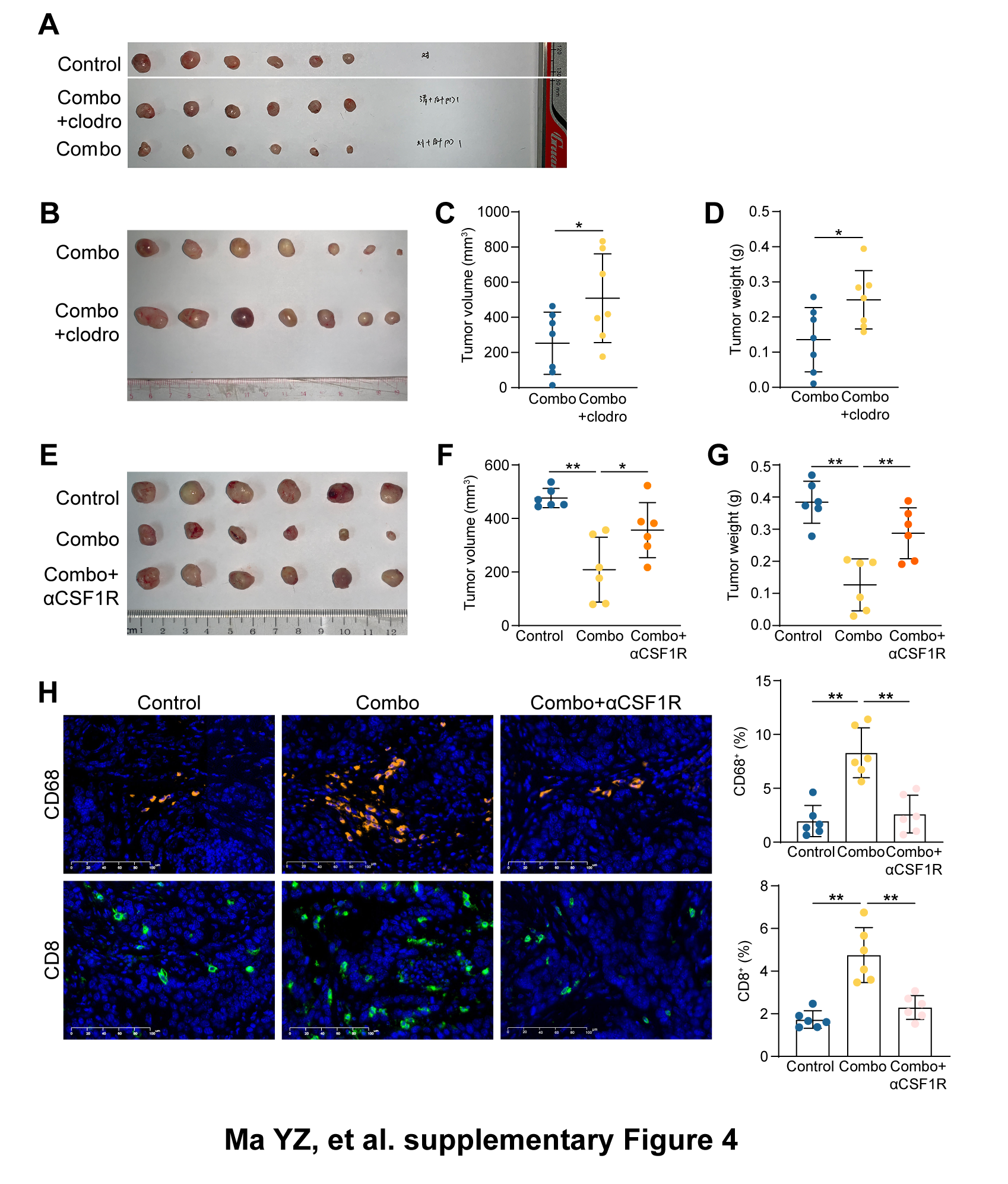


**Supplementary figure 4: Depletion of macrophages weakens the therapy effect of immunochemotherapy *in vivo*.**

**A)** Figure 3A with scale. **B)** Image of tumors formed on C57BL/6 mice that were treated with nab-PTX+anti-PD-1 (combo) and nab-PTX+anti-PD-1+clodronate (combo+clodro) (n=7 per group). Clodronate liposomes were administered one week after implanting tumors to C57BL/6 mice. **C-D**) Tumor volume (**C**) and tumor weight (**D**) of (**B**) were summarized (n=7 per group; Student’s *t*-test). **E)** Image of tumors formed on C57BL/6 mice that were treated with nab-PTX+anti-PD-1 (combo), nab-PTX+anti-PD-1+anti-CSF1R (combo+αCSF1R) and control (n=6 per group). **F-G**) Tumor volume (**F**) and tumor weight (**G**) of (**E**) were summarized (n=6 per group; Student’s *t*-test). **H)** Representative images and summary of IF staining of CD68 and CD8 in tumors of control, combo and combo+αCSF1R groups (E) (n=6 per group; Student’s *t*-test). (*, *P* < 0.05; **, *P* < 0.01; ***, *P* < 0.001)


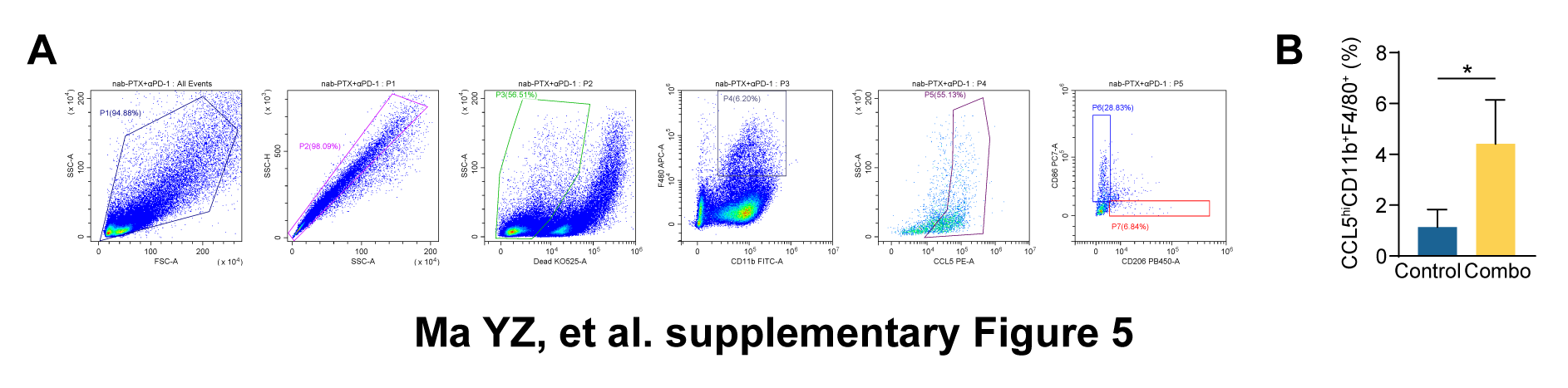


**Supplementary figure 5: The gating strategy depicted in Figure 4F and CCL5^hi^ macrophages in tumors.**

**A)** The gating strategy of Figure 4F. **B)** The proportion of CCL5^hi^ macrophages (CCL5^hi^CD11b^+^F4/80^+^) among viable cells isolated from tumors of combo (nab-PTX+anti-PD-1) and control groups (n=3 per group; *, *P* < 0.05; Student’s *t*-test). The proportion of CCL5^hi^CD11b^+^F4/80^+^ cells was determined based on all viable cells isolated from tumors.


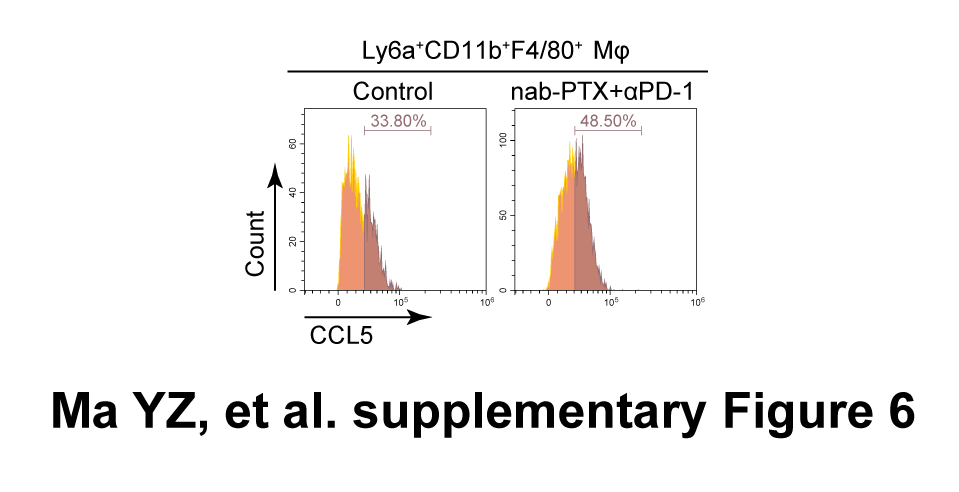


**Supplementary figure 6: Representative flow cytometry plots of CCL5 in Ly6a^+^CD11b^+^F4/80^+^ cells in Figure 5C.**

Representative flow cytometry plots of CCL5 in Ly6a^+^CD11b^+^F4/80^+^ cells in Figure 5C (n=3 per group; Student’s *t*-test).


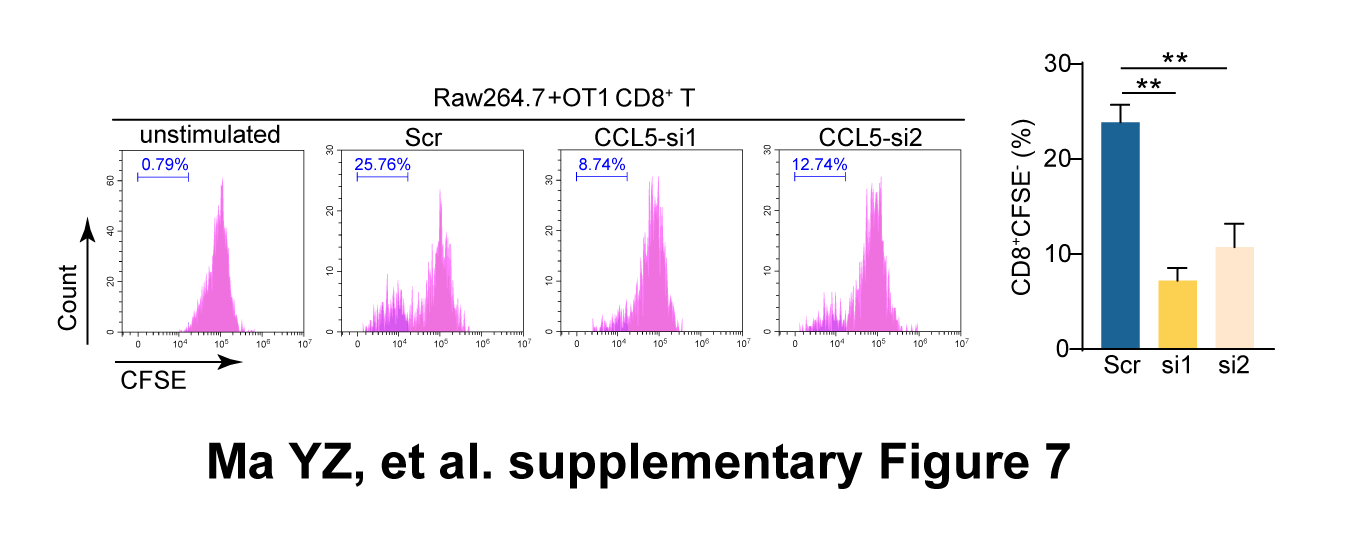


**Supplementary figure 7: The proliferation assay of CD8^+^ T cells derived from OT1 mice.**

CD8^+^ T cells derived from OT1 mice were co-cultured with RAW264.7 transfected with siRNAs targeting CCL5 and scramble control in the presence of SIINFEKL OVA peptide (100 nM) and IL-2 (50 ng/ml). Representative flow cytometry plots (*left*) and summary of CD8^+^CFSE^-^ cells ratio (*right*) (n=3 per group; Student’s *t*-test) (**, *P* < 0.01)


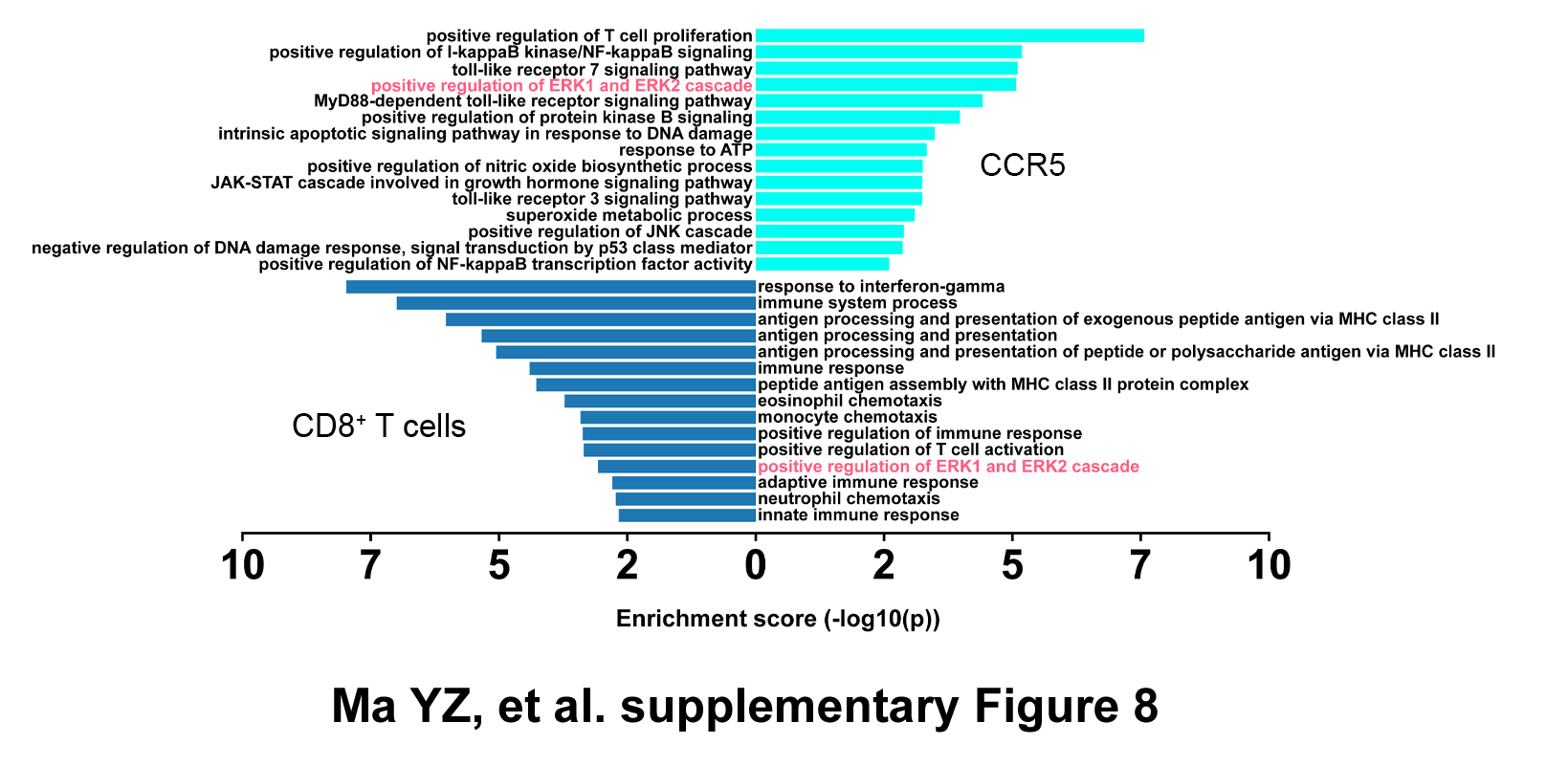


**Supplementary figure 8: Signaling pathways involved in CD8^+^ T cells and CCR5 functions.**

GO analysis results of DEGs of CD8^+^ T cell clusters between immunochemotherapy and control groups based on scRNA-seq data and main functions of CCR5 enriched in Coexpedia (https://www.coexpedia.org/).


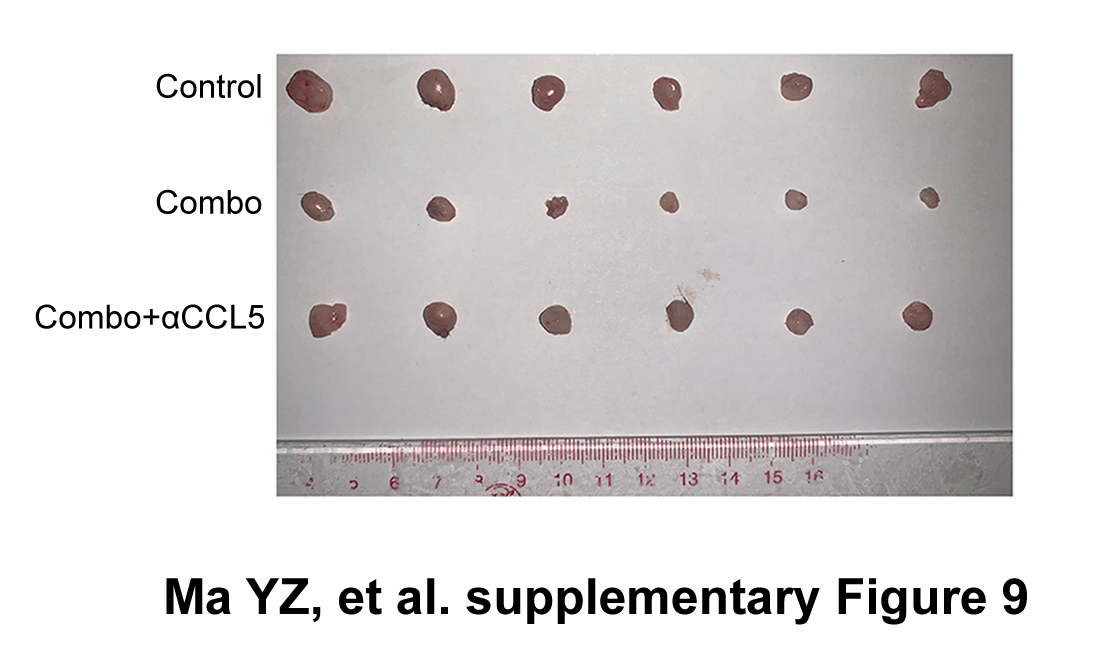


**Supplementary figure 9: Figure 6A with scale.**

Figure 6A with scale. (vehicle control; Combo: nab-PTX+anti-PD-1; combo+αCCL5: nab-PTX+anti-PD-1+anti-CCL5) (n=6 per group).


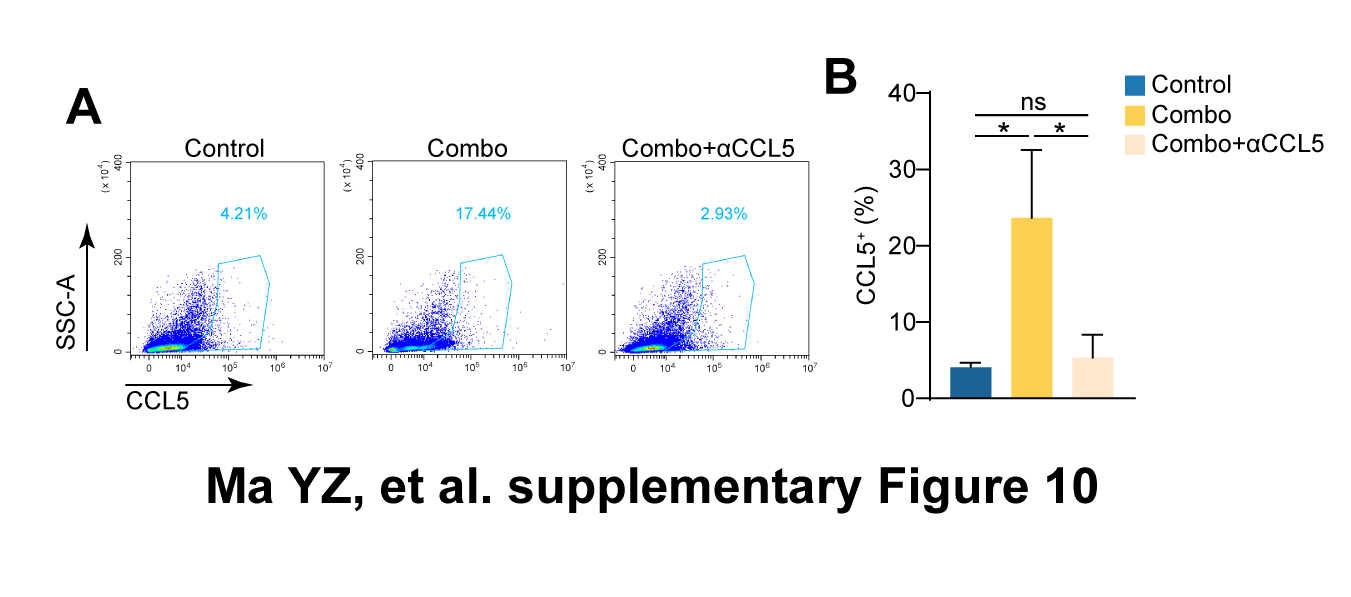


**Supplementary figure 10：The proportion of CCL5^+^ cells in the tumors of *in vivo* assay (Figure 6A).**

**A-B**) Representative flow cytometry plots **(A)** and summary of CCL5^+^ cells proportion **(B)** in the tumors in Figure 6A (n=3 per group; Student’s *t*-test). (*, *P* < 0.05; ns, not significant)


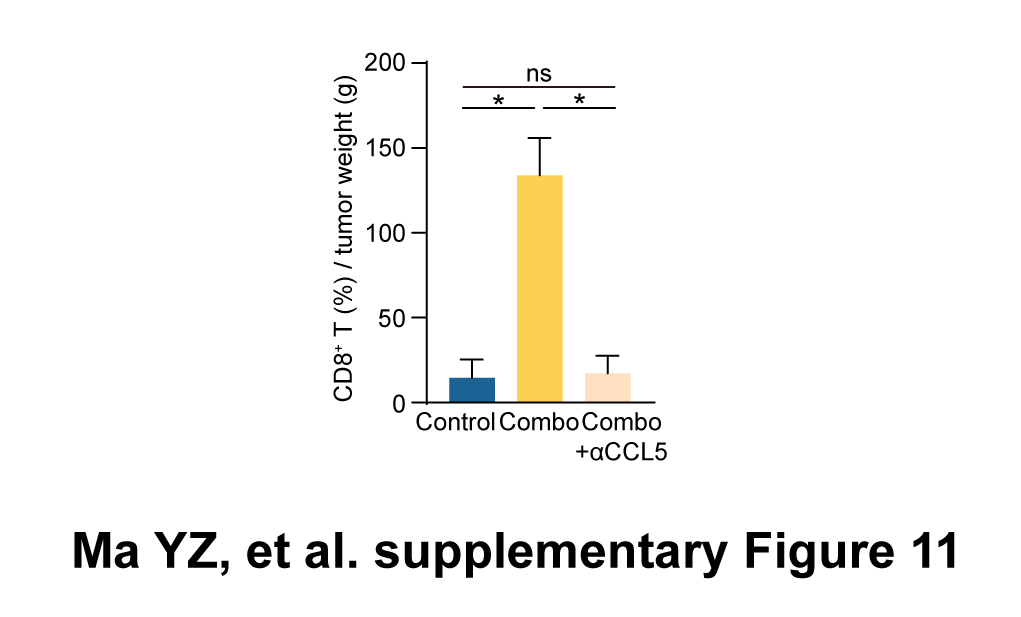


**Supplementary figure 11:** **The normalized infiltration of CD8^+^ T cells in the tumors (Figure 6E).**

Summary of CD8^+^ T cells infiltration (%) normalized by tumor weight (g) (Figure 6E) (n=3 per group; Student’s *t*-test). (*, *P* < 0.05; ns, not significant).


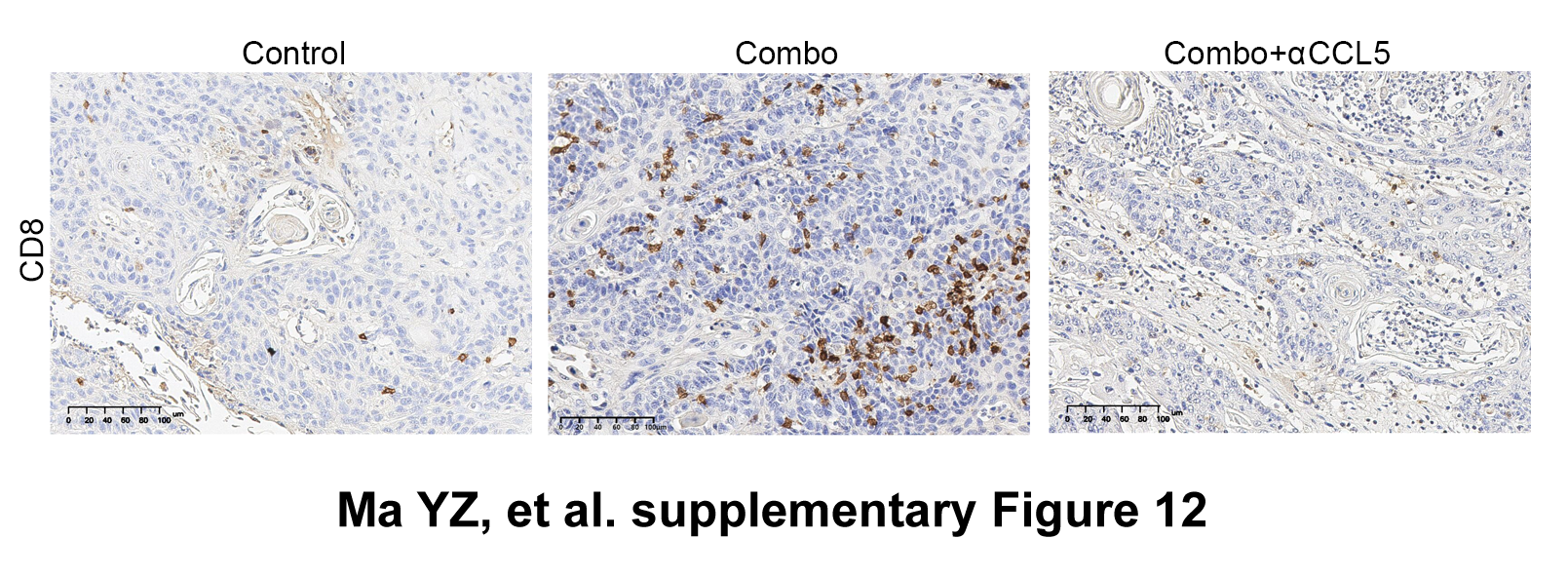


**Supplementary figure 12：IHC staining of CD8 in tumors of different experimental groups.**

Representative images of CD8 staining in tumors of the combo (nab-PTX+anti-PD-1), combo+anti-CCL5 (nab-PTX+anti-PD-1+anti-CCL5) and vehicle control groups.


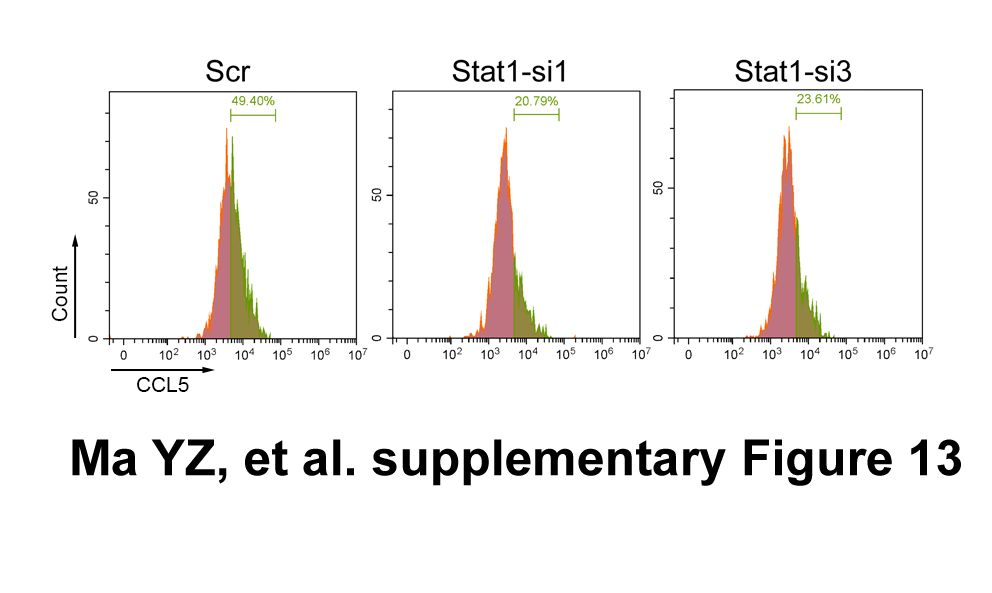


**Supplementary figure 13: Representative flow cytometry plots of MFI of CCL5 in RAW264.7 cells transfected with siRNAs targeting Stat1 or scramble control (Figure 7F).**

Representative flow cytometry plots of MFI of CCL5 in RAW264.7 cells transfected with siRNAs targeting Stat1 or scramble control (Figure 7F). (n=3 per group; Student’s *t*-test)


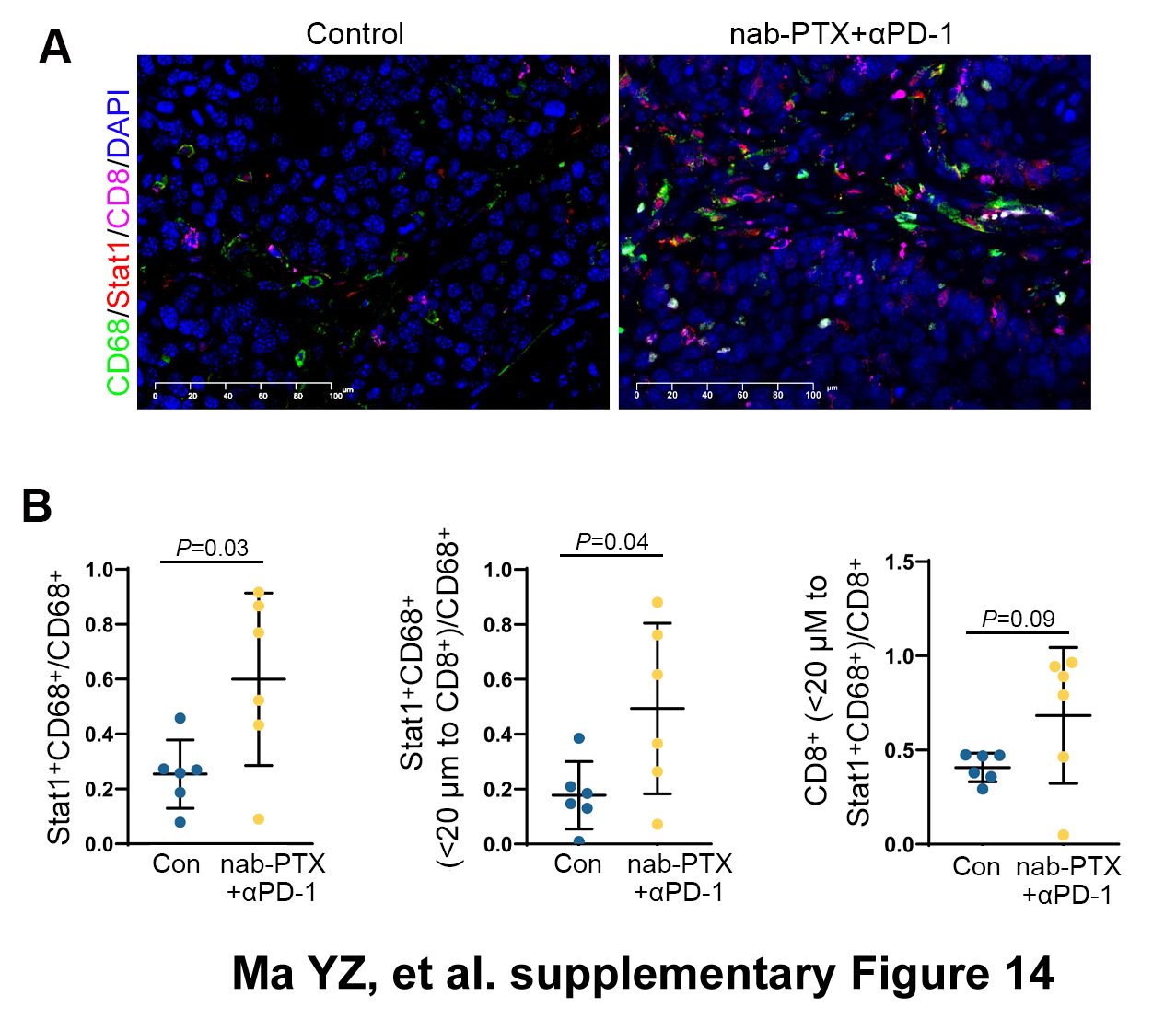


**Supplementary figure 14: mfIHC staining results in the tumors of immunochemotherapy and control groups.**

**A)** Representative pictures of mfIHC staining of CD68, Stat1 and CD8 in tumors of the immunochemotherapy (nab-PTX+anti-PD-1) and vehicle control groups. **B**) Summary of mfIHC results of (**A**). (n=6 per group; Student’s *t*-test)


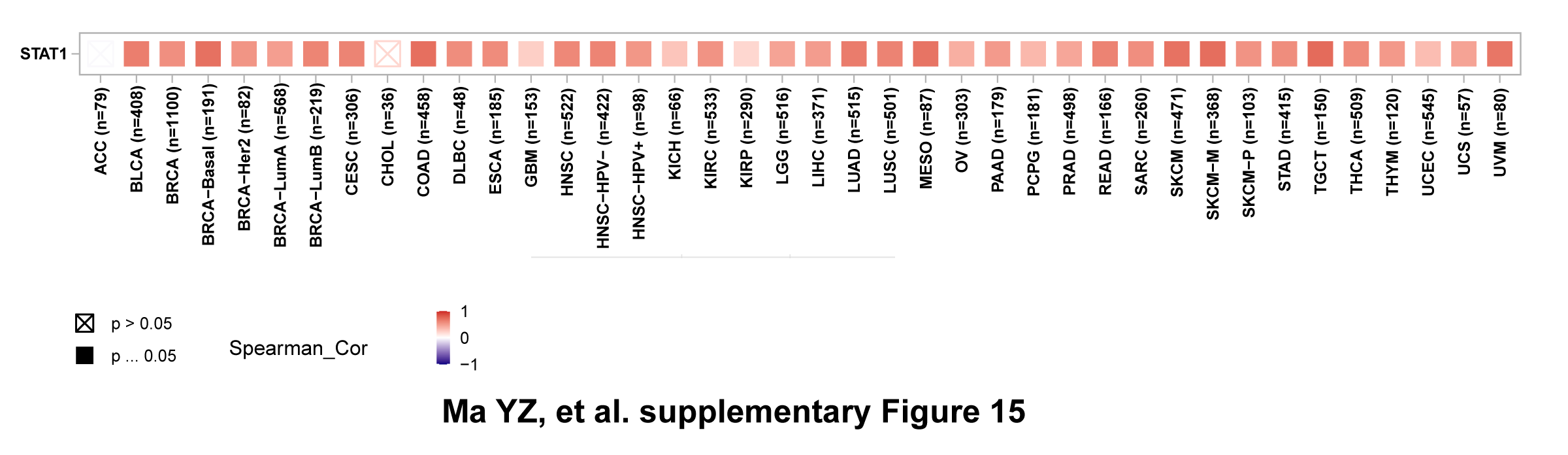


**Supplementary figure 15: The correlation between CCL5 and Stat1 in TCGA pan-cancer database.**

Heatmap showing the correlation between CCL5 and Stat1 in TCGA pan-cancer database calculated by TIMER 2.0.


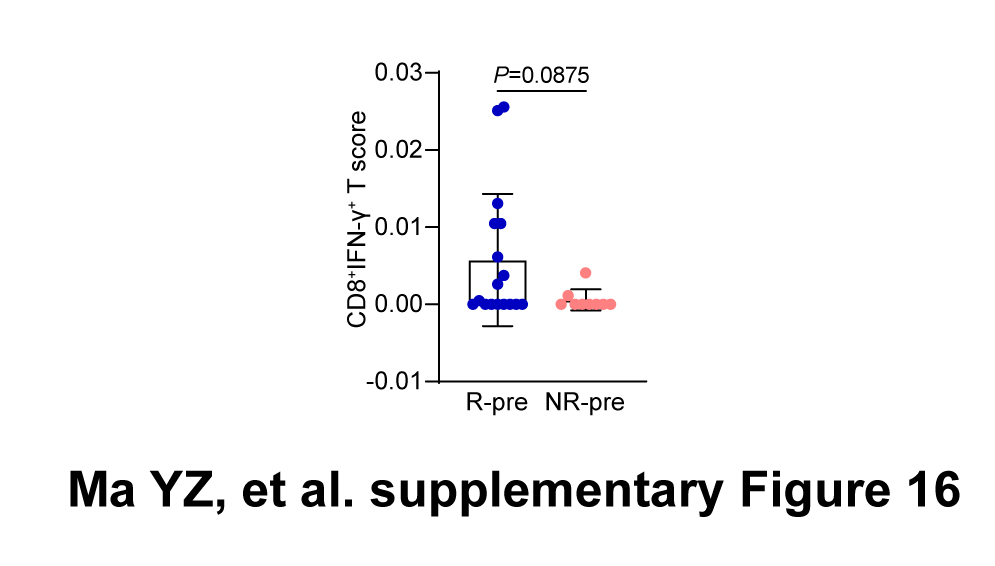


**Supplementary figure 16: CD8^+^IFNγ^+^ T cells score in SYSUCC cohort.**

A scatter plot was employed to visualize the score of CD8^+^IFNγ^+^ T cells in SYSUCC cohort (R-pre: n=17; NR-pre: n=9; Student’s *t*-test). Outliers beyond 1×standard deviation were removed prior to statistical analysis.


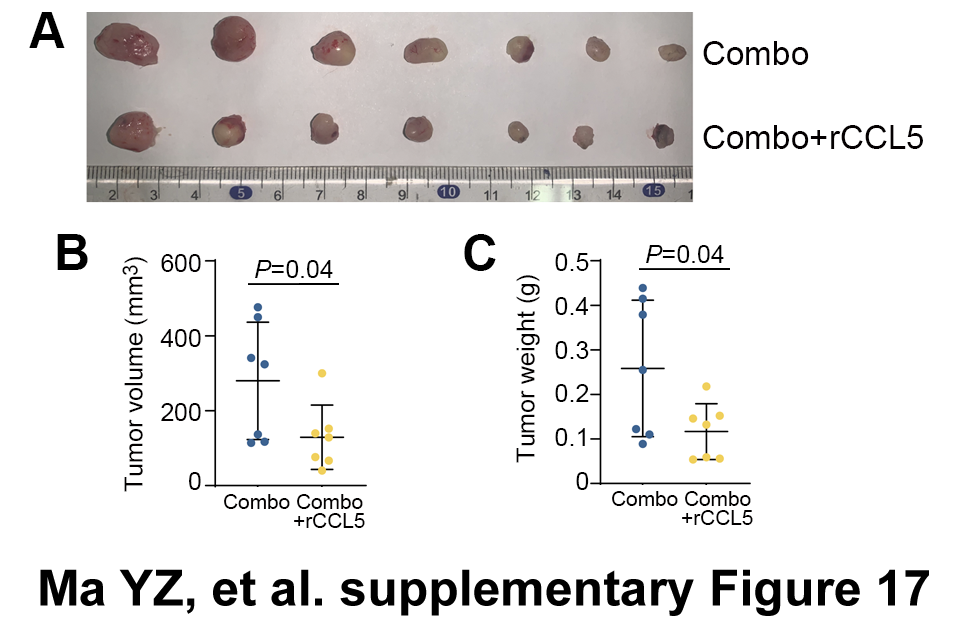


**Supplementary figure 17: Recombinant CCL5 protein improves the therapy effect of immunochemotherapy *in vivo*.**

**A)** The images of tumors formed in C57BL/6J mice receiving combo therapy (nab-PTX+anti-PD-1) or combo+recombinant CCL5 protein (rCCL5) (n=7 per group). **B-C**) Tumor volume (**B**) and tumor weight (**C**) were summarized (n=7 per group). (Student’s *t*-test).


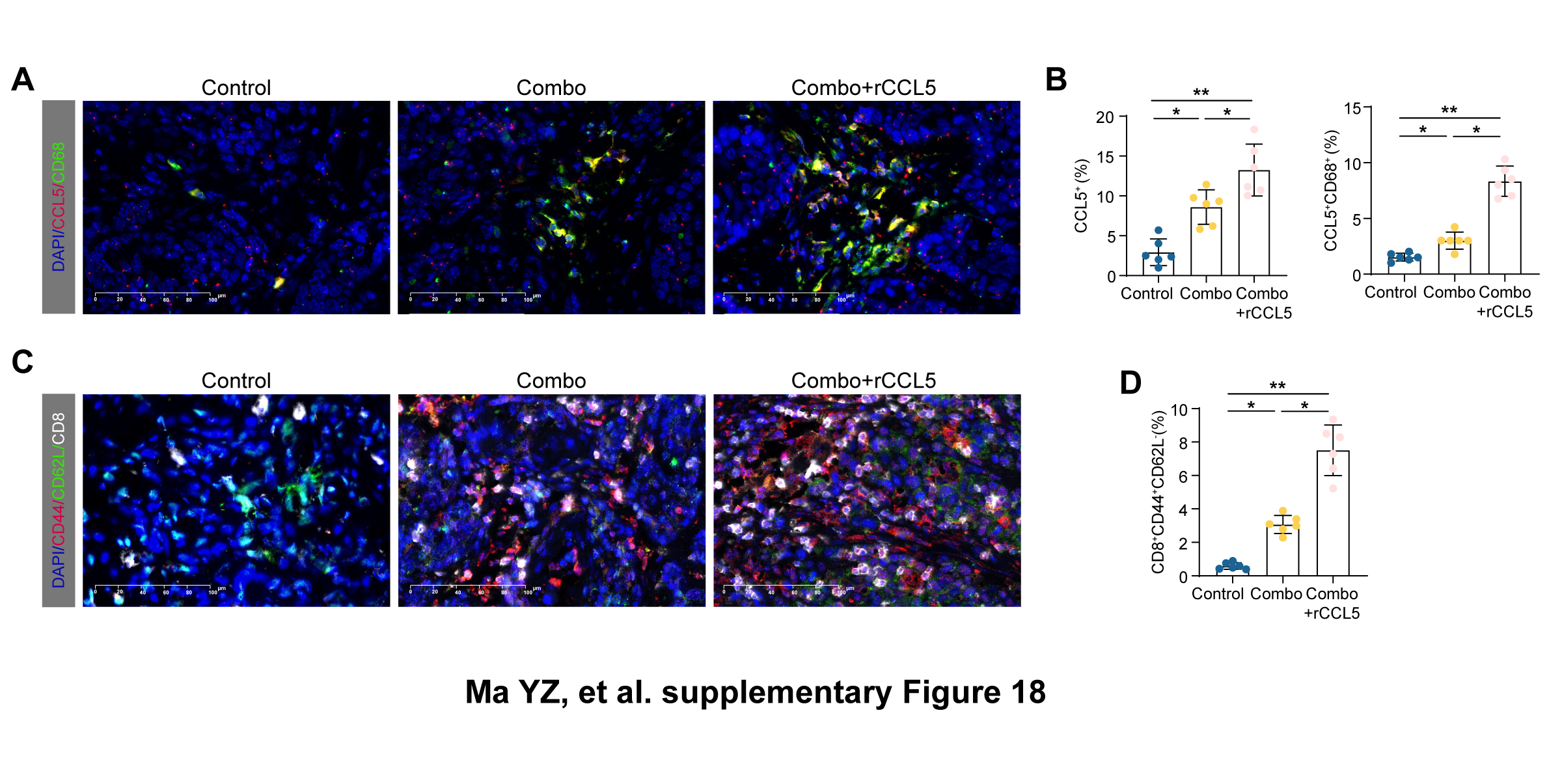


**Supplementary figure 18: mfIHC staining results in the tumors of *in vivo* assay (Figure 8J).**

**A)** Representative images of mfIHC staining of CD68 and CCL5 in the tumors of Figure 8J (n=6 per group). **B**) Summary of the proportions of CCL5^+^ cells or CCL5^+^CD68^+^ cells in (**A**) (n=6 per group; Student’s *t*-test). **C)** Representative pictures of mfIHC staining of CD8, CD44, CD62L in the tumors of Figure 8J. **D**) Summary of the proportion of CD8^+^CD44^+^CD62L^-^ cells in (**C**) (n=6 per group; Student’s *t*-test). (*, *P* < 0.05; **, *P*<0.01)


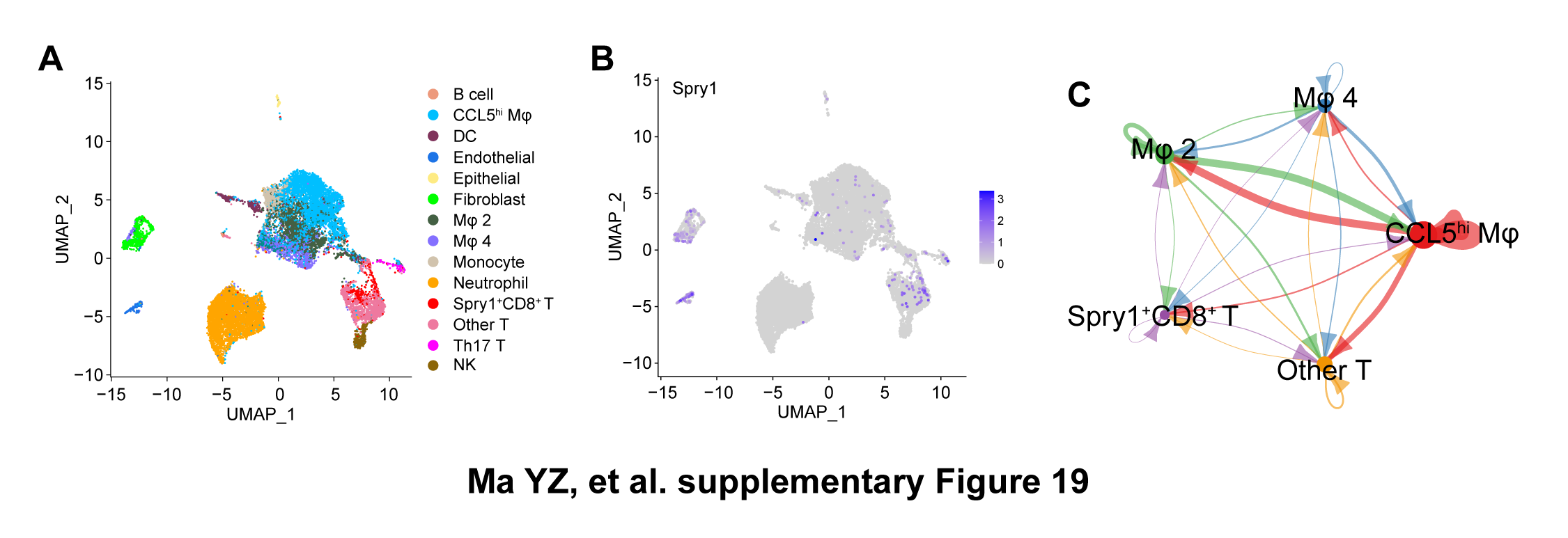


**Supplementary figure 19: Single-cell RNA sequencing analysis reveals** **a** **weak interaction between CCL5^hi^ macrophages and Spry1^+^CD8^+^ T cells.**

**A)** UMAP plot visualization showing all CD45^+^ cell clusters. **B**) UMAP plot visualization of Spry1 expression in cell clusters. **C**) Cell interaction strength between CCL5^hi^ macrophages and Spry1^+^CD8^+^ T cells and other cells.

| **The raw data for Figure 2B** | | | | | | | |
| --- | --- | --- | --- | --- | --- | --- | --- |
| Tumor Volumes (mm^3^) | | | | Tumor weight (g) | | | |
| Control | αPD-1 | nab-PTX | nab-PTX+αPD-1 | Control | αPD-1 | nab-PTX | nab-PTX+αPD-1 |
| 383.8464 | 253.8257 | 246.361 | 133.3984 | 0.262 | 0.198 | 0.15 | 0.082 |
| 290.7262 | 206.8823 | 284.333 | 162.0842 | 0.262 | 0.138 | 0.137 | 0.078 |
| 315.4484 | 249.8577 | 159.9802 | 89.875 | 0.259 | 0.126 | 0.117 | 0.069 |
| 312.6048 | 162.4211 | 191.016 | 73.41062 | 0.222 | 0.115 | 0.105 | 0.064 |
| 160.9457 | 188.7331 | 94.3433 | 31.07727 | 0.148 | 0.087 | 0.085 | 0.019 |
| 184.7689 | 145.9089 | 84.2948 | 20.32592 | 0.117 | 0.068 | 0.061 | 0.008 |

| **The raw data for Figure 3B** | | | | | |
| --- | --- | --- | --- | --- | --- |
| Tumor Volumes (mm^3^) | | | Tumor weight (g) | | |
| Control | Combo+clodro | Combo | Control | Combo+clodro | Combo |
| 341.5841 | 208.4025 | 156.9494 | 0.351 | 0.199 | 0.099 |
| 251.7881 | 167.5343 | 91.08821 | 0.267 | 0.183 | 0.073 |
| 222.775 | 143.338 | 64.31836 | 0.261 | 0.133 | 0.064 |
| 201.497 | 136.3469 | 56.31757 | 0.136 | 0.125 | 0.058 |
| 97.04448 | 131.0634 | 36.92326 | 0.103 | 0.101 | 0.031 |
| 82.38067 | 125.5954 | 30.33498 | 0.102 | 0.094 | 0.025 |

| **The raw data for Figure 6B** | | | | | |
| --- | --- | --- | --- | --- | --- |
| Tumor Volumes (mm^3^) | | | Tumor weight (g) | | |
| Control | Combo | Combo+αCCL5 | Control | Combo | Combo+αCCL5 |
| 882.75 | 285.2543 | 1289.403 | 0.2634 | 0.1903 | 0.3123 |
| 654.6065 | 155.5786 | 832.4544 | 0.255 | 0.147 | 0.2755 |
| 550.4603 | 141.0953 | 557.329 | 0.2486 | 0.1317 | 0.1816 |
| 295.2232 | 130.3271 | 226.9654 | 0.2 | 0.1044 | 0.1718 |
| 108.7054 | 34.04061 | 218.3392 | 0.1099 | 0.0423 | 0.1383 |
| 93.28236 | 17.72682 | 193.854 | 0.0992 | 0.0224 | 0.1164 |

| **The raw data for Figure 6E** | | | |
| --- | --- | --- | --- |
|  | CD8^+^ T (%) | Tumor tissue volumes (mm^3^) | CD8^+^ T (%)/Tumor tissue volumes (cm^3^) |
| Control 1 | 3.56 | 441.375 | 8.065703767 |
| Control 2 | 0.41 | 54.35268 | 7.54332629 |
| Control 3 | 0.45 | 46.64118 | 9.648126398 |
| Combo 1 | 10.32 | 77.7893 | 132.6660608 |
| Combo 2 | 10 | 70.54765 | 141.748166 |
| Combo 3 | 10.41 | 142.62715 | 72.98750624 |
| Combo+αCCL5 1 | 0.31 | 96.927 | 3.198283244 |
| Combo+αCCL5 2 | 3.41 | 644.7015 | 5.28926953 |
| Combo+αCCL5 3 | 3.38 | 416.2272 | 8.120564922 |

| **The raw data for Figure 8K** | | | | | |
| --- | --- | --- | --- | --- | --- |
| Tumor Volumes (mm^3^) | | | Tumor weight (g) | | |
| Control | Combo | Combo+rCCL5 | Control | Combo | Combo+rCCL5 |
| 537.4979 | 430.2437 | 96.26954 | 0.282 | 0.19 | 0.117 |
| 533.6878 | 246.4517 | 79.57055 | 0.277 | 0.13 | 0.095 |
| 517.6846 | 223.9526 | 68.33286 | 0.243 | 0.122 | 0.085 |
| 503.1114 | 203.7777 | 65.97192 | 0.212 | 0.134 | 0.066 |
| 498.9204 | 168.3828 | 32.56496 | 0.202 | 0.117 | 0.011 |
| 493.8029 | 81.26554 | 144.2613 | 0.21 | 0.051 | 0.008 |

| **The raw data for Supplementary Figure 4C-D** | | | |
| --- | --- | --- | --- |
| Tumor Volumes (mm^3^) | | Tumor weight (g) | |
| Combo | Combo+clodro | Combo | Combo+clodro |
| 463.3366 | 176.1894 | 0.0105 | 0.3942 |
| 413.0565 | 832.5145 | 0.0424 | 0.2904 |
| 118.1882 | 296.234 | 0.0929 | 0.2541 |
| 367.1823 | 647.4884 | 0.14 | 0.1575 |
| 306.0437 | 395.2268 | 0.1925 | 0.2841 |
| 87.98297 | 793.9754 | 0.2132 | 0.1902 |
| 13.83384 | 417.7572 | 0.2571 | 0.1735 |

| **The raw data for Supplementary Figure 4F-G** | | | | | |
| --- | --- | --- | --- | --- | --- |
| Tumor Volumes (mm^3^) | | | Tumor weight (g) | | |
| Control | Combo | Combo+αCSF1R | Control | Combo | Combo+αCSF1R |
| 536.1708 | 356.8803 | 522.6417 | 0.468 | 0.205 | 0.388 |
| 502.2892 | 341.02 | 388.3225 | 0.435 | 0.197 | 0.348 |
| 470.4993 | 217.1615 | 382.016 | 0.385 | 0.194 | 0.315 |
| 451.9988 | 177.2122 | 332.3943 | 0.373 | 0.088 | 0.28 |
| 451.98 | 82.24031 | 296.5959 | 0.365 | 0.047 | 0.201 |
| 444.925 | 79.29962 | 217.2766 | 0.278 | 0.03 | 0.191 |

| **The raw data for Supplementary Figure 11** | | | |
| --- | --- | --- | --- |
|  | CD8^+^ T (%) | Tumor tissue weight (g) | CD8^+^ T (%)/Tumor tissue weight (g) |
| Control 1 | 3.56 | 0.1317 | 27.03113136 |
| Control 2 | 0.41 | 0.05494 | 7.462686567 |
| Control 3 | 0.45 | 0.049615 | 9.069837751 |
| Combo 1 | 10.32 | 0.0735 | 140.4081633 |
| Combo 2 | 10 | 0.06585 | 151.8602885 |
| Combo 3 | 10.41 | 0.09515 | 109.4062007 |
| Combo+αCCL5 1 | 0.31 | 0.0582 | 5.326460481 |
| Combo+αCCL5 2 | 3.41 | 0.15615 | 21.8379763 |
| Combo+αCCL5 3 | 3.38 | 0.13775 | 24.53720508 |

| **The raw data for Supplementary Figure 17** | | | |
| --- | --- | --- | --- |
| Tumor Volumes (mm^3^) | | Tumor weight (g) | |
| Combo | Combo+rCCL5 | Combo | Combo+rCCL5 |
| 476.2201 | 300.1655 | 0.415 | 0.218 |
| 449.7658 | 152.2155 | 0.439 | 0.146 |
| 340.9552 | 139.7076 | 0.379 | 0.152 |
| 323.8615 | 128.5358 | 0.255 | 0.056 |
| 136.4033 | 40.05647 | 0.122 | 0.054 |
| 117.3679 | 76.25667 | 0.11 | 0.059 |
| 114.2291 | 66.89177 | 0.089 | 0.132 |

| **Clinical information of patients** | | | | | | | | | | | | |  |
| --- | --- | --- | --- | --- | --- | --- | --- | --- | --- | --- | --- | --- | --- |
| **Patient ID** | **Gender** | **Age(yrs):** | **Location** | **differentiation** | **Smoking** | **Drinking** | **Response** | **T stage** | **N stage** | **Clinical stage** | **mfIHC** | |  |
| Pt1 | M | 56 | Middle | Moderate | Yes | Yes | NR | 3 | 2 | III | CD8/CD68/panCK |  | |
| Pt2 | M | 52 | Lower | Moderate | Yes | Yes | NR | 3 | 2 | III | CD8/CD68/panCK |  | |
| Pt3 | M | 63 | Middle | Moderate | Yes | Yes | NR | 3 | 2 | III | CD8/CD68/panCK |  | |
| Pt4 | F | 50 | Middle | Poor | No | No | NR | 2 | 2 | III | CD8/CD68/panCK | STAT1/CD8/CD68/panCK | |
| Pt5 | M | 69 | Middle | Well | Yes | Yes | NR | 2 | 1 | II | CD8/CD68/panCK | STAT1/CD8/CD68/panCK | |
| Pt6 | M | 66 | Middle | Moderate | Yes | Yes | NR | 3 | 2 | III | CD8/CD68/panCK |  | |
| Pt7 | M | 50 | Lower | Moderate | Yes | No | NR | 3 | 1 | III | CD8/CD68/panCK | STAT1/CD8/CD68/panCK | |
| Pt8 | M | 57 | Middle | Moderate | Yes | No | NR | 3 | 1 | III | CD8/CD68/panCK | STAT1/CD8/CD68/panCK | |
| Pt9 | M | 62 | Lower | Poor | Yes | Yes | NR | 3 | 1 | III | CD8/CD68/panCK |  | |
| Pt10 | F | 67 | Middle | Moderate | No | No | MPR | 2 | 0 | III | CD8/CD68/panCK |  | |
| Pt11 | M | 54 | Middle | Moderate | Yes | Yes | MPR | 3 | 2 | III | CD8/CD68/panCK |  | |
| Pt12 | M | 58 | Middle | Moderate | No | No | MPR | 1 | 0 | II | CD8/CD68/panCK | STAT1/CD8/CD68/panCK | |
| Pt13 | M | 60 | Lower | Poor | Yes | Yes | MPR | 1 | 0 | II | CD8/CD68/panCK |  | |
| Pt14 | M | 57 | Middle | Moderate | No | No | MPR | 3 | 2 | III | CD8/CD68/panCK | STAT1/CD8/CD68/panCK | |
| Pt15 | M | 52 | Middle | Moderate | Yes | No | MPR | 3 | 0 | I | CD8/CD68/panCK |  | |
| Pt16 | M | 63 | Middle | Moderate | Yes | No | MPR | 2 | 1 | II | CD8/CD68/panCK |  | |
| Pt17 | F | 70 | Middle | Moderate | No | No | MPR | 3 | 1 | III | CD8/CD68/panCK | STAT1/CD8/CD68/panCK | |
| Pt18 | M | 49 | Middle | Moderate | Yes | Yes | pCR | 3 | 2 | III | CD8/CD68/panCK | STAT1/CD8/CD68/panCK | |
| Pt19 | M | 49 | Middle | Moderate | Yes | Yes | pCR | 3 | 2 | III | CD8/CD68/panCK |  | |
| Pt20 | M | 47 | Middle | Moderate | Yes | Yes | pCR | 2 | 1 | II | CD8/CD68/panCK |  | |
| Pt21 | F | 63 | Middle | Moderate | No | No | pCR | 3 | 2 | III | CD8/CD68/panCK | STAT1/CD8/CD68/panCK | |
| Pt22 | M | 51 | Middle | Moderate | Yes | Yes | pCR | 3 | 1 | III | CD8/CD68/panCK |  | |
| Pt23 | M | 69 | Middle | Moderate | Yes | No | pCR | 2 | 1 | II | CD8/CD68/panCK | STAT1/CD8/CD68/panCK | |
| Pt24 | M | 66 | Middle | Moderate | No | No | pCR | 3 | 1 | III | CD8/CD68/panCK |  | |
| Pt25 | F | 66 | Middle | Moderate | No | No | pCR | 3 | 2 | III | CD8/CD68/panCK |  | |
| Pt26 | M | 61 | Lower | Poor | Yes | Yes | NR | 3 | 0 | II |  | STAT1/CD8/CD68/panCK | |
| Pt27 | M | 57 | Lower | Moderate | No | No | pCR | 3 | 1 | IV |  | STAT1/CD8/CD69/panCK | |
| Pt28 | M | 58 | Lower | Moderate | Yes | No | pCR | 1 | 2 | IV |  | STAT1/CD8/CD70/panCK | |
| Pt29 | M | 53 | Middle/Lower | Well | Yes | No | MPR | 3 | 2 | III |  | STAT1/CD8/CD71/panCK | |
| Pt30 | M | 69 | Lower | Poor | Yes | No | NR | 2 | 2 | III |  | STAT1/CD8/CD72/panCK | |
| Pt31 | M | 55 | Middle/Lower | Moderate | No | Yes | NR | 4 | 0 | III |  | STAT1/CD8/CD73/panCK | |
| Pt32 | M | 60 | Upper | Squamous cell carcinoma | Yes | No | pCR | 2 | 1 | II |  | STAT1/CD8/CD74/panCK | |
| Pt33 | M | 48 | Lower | Squamous cell carcinoma | No | No | MPR | 3 | 1 | III |  | STAT1/CD8/CD75/panCK | |
| Pt34 | M | 64 | Lower | Moderate | Yes | Yes | NR | 2 | 1 | III |  | STAT1/CD8/CD76/panCK | |
| Pt35 | M | 52 | Middle/Lower | Moderate | No | No | MPR | 2 | 3 | III |  | STAT1/CD8/CD77/panCK | |
| Pt36 | M | 68 | Middle | Squamous cell carcinoma | No | No | NR | 3 | 2 | III |  | STAT1/CD8/CD78/panCK | |
| Pt37 | M | 65 | Middle | Moderate | Yes | Yes | MPR | 3 | 3 | IV |  | STAT1/CD8/CD68/panCK | |
| Pt38 | F | 44 | Middle | Moderate | No | No | MPR | 2 | 1 | II |  | STAT1/CD8/CD68/panCK | |
| Pt39 | M | 63 | Middle | Poor | Yes | Yes | NR | 3 | 0 | II |  | STAT1/CD8/CD68/panCK | |
| Pt40 | F | 63 | Middle | Poor | No | No | pCR | 3 | 1 | III |  | STAT1/CD8/CD68/panCK | |
| Pt41 | M | 56 | Middle | Moderate | Yes | Yes | pCR | 3 | 2 | III |  | STAT1/CD8/CD68/panCK | |
| Pt42 | M | 46 | Lower | Moderate | No | No | pCR | 3 | 2 | III |  | STAT1/CD8/CD68/panCK | |
